# Arginine methylation and ubiquitylation crosstalk controls DNA end-resection and homologous recombination repair

**DOI:** 10.1038/s41467-021-26413-6

**Published:** 2021-11-02

**Authors:** Maria Pilar Sanchez-Bailon, Soo-Youn Choi, Elizabeth R. Dufficy, Karan Sharma, Gavin S. McNee, Emma Gunnell, Kelly Chiang, Debashish Sahay, Sarah Maslen, Grant S. Stewart, J. Mark Skehel, Ingrid Dreveny, Clare C. Davies

**Affiliations:** 1 Institute of Cancer and Genomic Sciences, University of Birmingham, Birmingham, UK; 2 Biodiscovery Institute, School of Pharmacy, University of Nottingham, Nottingham, UK; 3 MRC Laboratory of Molecular Biology, Cambridge, UK; 4 Present Address: Max Delbrück Center for Molecular Medicine, Berlin, Germany; 5 Present Address: The Francis Crick Institute, London, UK

**Keywords:** DNA repair enzymes, Methylation, Ubiquitylation, Homologous recombination

## Abstract

Cross-talk between distinct protein post-translational modifications is critical for an effective DNA damage response. Arginine methylation plays an important role in maintaining genome stability, but how this modification integrates with other enzymatic activities is largely unknown. Here, we identify the deubiquitylating enzyme USP11 as a previously uncharacterised PRMT1 substrate, and demonstrate that the methylation of USP11 promotes DNA end-resection and the repair of DNA double strand breaks (DSB) by homologous recombination (HR), an event that is independent from another USP11-HR activity, the deubiquitylation of PALB2. We also show that PRMT1 is a ubiquitylated protein that it is targeted for deubiquitylation by USP11, which regulates the ability of PRMT1 to bind to and methylate MRE11. Taken together, our findings reveal a specific role for USP11 during the early stages of DSB repair, which is mediated through its ability to regulate the activity of the PRMT1-MRE11 pathway.

## Abstract (web summary)

Post-translational modifications are critical for regulating the DNA damage response. Here, the authors identify a methylation-deubiquitination crosstalk between methyltransferase PRMT1 and deubiquitinase USP11, showing that the enzymes regulate each other’s functions in DNA repair.

## Introduction

The integrity of the mammalian genome is constantly being challenged by a plethora of endogenous and exogenous stresses that must be repaired to maintain genome integrity. One of the most deleterious forms of DNA damage is the double-strand break (DSB), which if left unrepaired can lead to mutagenic events, including loss of genetic material and chromosomal translocations, that predispose individuals to pathological conditions including cancer and neurological dysfunction. DSB repair is coordinated by two evolutionally conserved but mechanistically distinct pathways: non-homologous end-joining (NHEJ) and homologous recombination (HR). NHEJ occurs throughout all stages of the cell cycle, requires minimal processing of the break ends and is rapid, but considered error-prone. In contrast, HR is error-free but requires extensive DNA end-resection to generate sufficient ssDNA to allow invasion of the sister chromatid. As such, this mechanism of repair only occurs during the late S/G2 phase of the cell cycle^1^.

The process of DSB repair, including damage sensing, the choice of repair pathway, and the termination of the repair response, is driven by a plethora of enzymes, including nucleases, polymerases, kinases, phosphatases, methyltransferases and E3 ubiquitin ligases. As such, these activities have to be tightly regulated to mitigate any inappropriate action that could be detrimental to the repair process itself. A key mechanism by which this is achieved is through protein post-translational modifications (PTMs), with protein phosphorylation and ubiquitylation being the best characterised^2^. For example, early sensing and recruitment of the MRE11–Rad50–NBS1 (MRN) complex to break ends leads to the recruitment and activation of the principal effector kinase, ATM^3^. ATM phosphorylates numerous proteins, including H2AX on serine 139 (γH2AX) at sites of damaged chromatin, which in turn is recognised by the BRCT domains of MDC1, allowing amplification in the ATM-dependent DNA damage response (DDR)^4,5^. ATM also phosphorylates a series of TQXF motifs on MDC1, which are recognised by the FHA domain of the E3 ubiquitin ligase RNF8. RNF8 ubiquitylates many proteins, including linker histone H1, which is then recognised by the UMI/MIU domains of RNF168. This triggers the ubiquitylation of H2A and H2AX by RNF168, which in turn promotes recruitment of anti-resection factor 53BP1 to damaged chromatin^6–11^.

Whilst the importance of phosphorylation and ubiquitylation is clear, one protein modification that we know very little regarding its role in regulating DNA repair is arginine methylation. Protein arginine methyltransferases (PRMTs) catalyse mono and dimethylation of the guanidino group of arginine residues using S-adenosyl methionine (SAM) as a cofactor, with dimethylation occurring either symmetrically (two methyl groups conjugated to adjacent nitrogens of the guanidino group) or asymmetrically (both methyl groups conjugated to the same nitrogen). The predominant PRMT family member, PRMT1, is responsible for the majority of asymmetric dimethylation in mammals^12^, and is known to be required for maintaining genome stability; mouse embryonic fibroblasts lacking *Prmt1* exhibit increased levels of spontaneous DNA damage, polyploidy, chromosomal instability and checkpoint defects after DNA damage^13^. General mechanistic insight into the role of PRMT1 in DNA repair is largely lacking but appears to involve both the direct methylation of DNA repair proteins and epigenetically induced gene expression changes^14–16^. One of the first DNA repair proteins shown to be methylated by PRMT1 was MRE11^14,17,18^. MRE11 is an integral component of the MRN complex and, by possessing both endo- and exonuclease activity, is a key mediator of DNA end-resection, a process known to commit a cell to DSB repair via HR. The endonuclease function of MRE11 induces a DNA nick several hundred basepairs away from the DSB, which enables the 3′–5′ exonuclease activity, in association with CtIP, to extend the nick towards the broken DNA ends (termed “short-range” end-resection). This then acts as a platform for long-range resection (several kilobases long) catalysed by DNA2, EXO1 and BLM. The resultant ssDNA is initially coated with replication protein A (RPA), which is then displaced by RAD51 to allow strand invasion and recombination^19^. PRMT1 specifically methylates MRE11 within its C-terminal glycine-rich arginine-rich (GAR) domain^14^. Mutation of the nine arginines within the GAR domain to lysine compromises the 3′–5′ exonuclease activity of MRE11 and disrupts activation of the intra-S-phase DNA checkpoint^14,17^. Consistent with the importance of MRE11 methylation by PRMT1, cells from mice expressing a form of Mre11 that cannot be methylated by Prmt1 are hypersensitive to IR, exhibit increased chromosomal instability, loss of DNA damage checkpoint activation and a failure to recruit RPA and Rad51 to DNA break ends^20^. PRMT1-mediated methylation of MRE11 appears to occur in a regulated manner as the T-cell specific transcription factor GFI1 is required for an effective DDR and functions in part as a PRMT1 cofactor increasing MRE11 methylation^18^.

Since PRMT1 is the major enzyme that catalyses cellular methylation and preferentially asymmetrically dimethylated arginine residues embedded within the RG/RGG motif^12,21^, PRMT1 substrate identification has largely been carried out through affinity purification using antibodies that recognise asymmetrically dimethylated RG/RGG motifs^22,23^. Whilst this approach has identified numerous PRMT1 substrates, including MRE11^23^, it is clear that some PRMT1 substrates are methylated within non-consensus sites^24–26^. Hence, alternative proteomic approaches are required to fully elucidate the substrate repertoire of PRMT1.

To address this, we conducted mass spectrometry analysis of PRMT1 interacting proteins and identified the deubiquitylating enzyme (DUB) USP11 as a PRMT1 substrate. USP11 is a member of the ubiquitin-specific protease (USP) subfamily that has previously been implicated in regulating HR through modulating the formation of the BRCA1–PALB2–BRCA2 complex to enable RAD51 loading^27^. Correspondingly, depletion of USP11 sensitises cells to genotoxic agents that induce DNA lesions that require HR for repair, for example, PARP inhibitors and camptothecin^28,29^. Here, we show that PRMT1-mediated methylation of USP11 modulates its ability to promote HR. We also find that USP11 deubiquitylates PRMT1 and that this correlates with an increase in the methylation of the PRMT1 substrate MRE11. Together, this influences DNA end-resection and HR-mediated repair of DSBs. Given that USP11 expression levels are upregulated in the S/G2 phase^27^, our findings suggest a mechanism by which PRMT1-mediated activation of MRE11 nuclease activity is regulated within S/G2 cells to ensure proper cell cycle-dependent control of DNA end-resection.

## Results

### USP11 is methylated by PRMT1

The majority of arginine-methyl proteomic datasets employ mono-methyl or di-methyl antibodies raised against RG/RGG motifs for affinity purification, thereby enriching for methyl-RG/RG proteins. Since 29% of methylated arginines are outside of the consensus RG/RGG motif^22^, to discover PRMT1 substrates without biasing substrate identification we conducted Flag-PRMT1 purification and mass spectrometry analysis (Supplementary Fig. 1a Supplementary Data 1). This approach identified a number of established PRMT1 substrates but also identified the ubiquitin-specific protease USP11 as a yet-to-be described PRMT1-interacting protein. Given the importance of ubiquitylation in regulating the DDR and that USP11 has been implicated in this process^27,28,30–34^, we decided to investigate the significance of the PRMT1/USP11 interaction further.

**Fig. 1:**
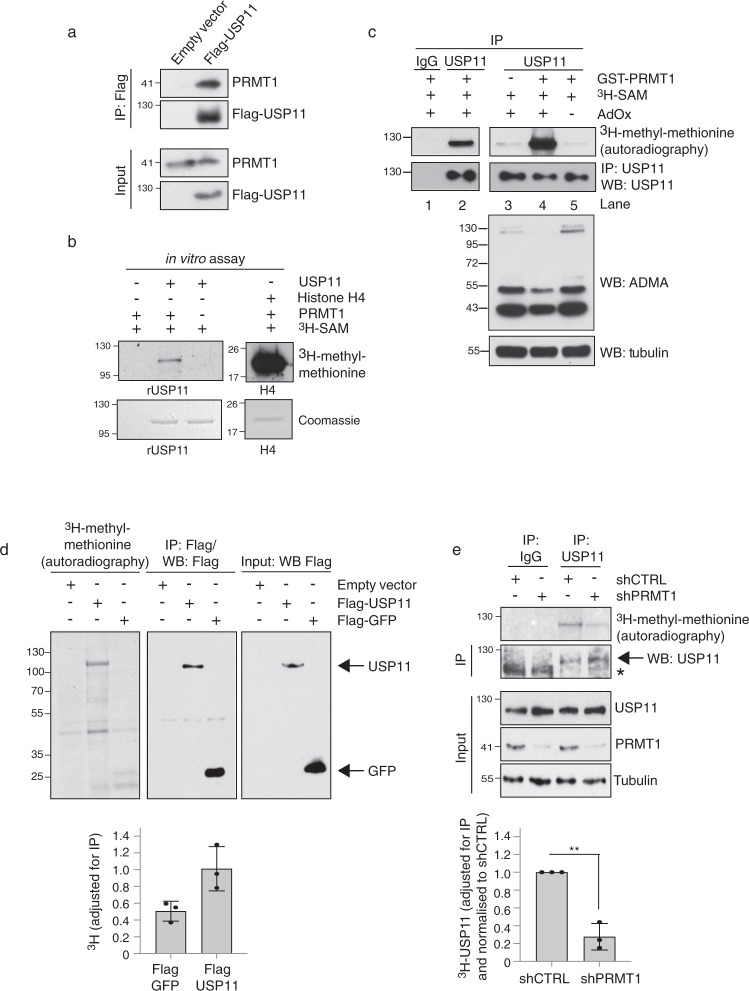
PRMT1 interacts with and methylates USP11. **a** Flag-tagged USP11 co-immunoprecipitates endogenous PRMT1 in MCF7 cells. Representative image from three independent repeats. **b** Recombinant USP11 is methylated by recombinant PRMT1 as determined by in vitro methylation assay. Methylation of histone H4 serves as a positive control. Representative image from three independent repeats. **c** Recombinant PRMT1 methylates endogenous USP11 isolated from 293T cells as determined by in vitro methylation assay. Incubation with 20 µM AdOx for 24 h generates hypomethylated substrate appropriate for enzymatic modification (compare lanes 4 and 5). Representative image from three independent repeats. **d** USP11 is methylated in vivo. 293T cells were transfected with the indicated plasmids and de novo protein synthesis inhibited by CAM/CHX treatment. Following [^3^H]-methyl-methionine labelling, Flag-USP11 was immunoprecipitated and incorporated methyl groups detected by SDS-PAGE and autoradiography. Flag-GFP serves as a control for protein synthesis inhibition. The graph represents quantification of methyl incorporation adjusted for the amount of protein immunoprecipitation (mean ± SD; *n* = 3 biologically independent experiments). **e** In vivo endogenous USP11 methylation is PRMT1-dependent. MCF7 cells stably expressing shPRMT1 were treated with CAM/CHX, labelled with [^3^H]-methyl-methionine, followed by endogenous USP11 immunoprecipitation. The incorporation of methyl groups was determined by SDS-PAGE and autoradiography. Asterisk represents non-specific protein immunoprecipitating with IgG control. Graph represents quantification of methyl-incorporation adjusted for USP11 immunoprecipitation and normalised to MCF7-shCTRL (mean ± SD; *n* = 3 biological independent experiments; **p = 0.00108 (Student’s *t* test, two-sided, equal variance). Uncropped blots and processed graphical data are provided as a Source Data file.

We found that ectopically expressed USP11 co-immunoprecipitated endogenous PRMT1 in cells (Fig. 1a) and that this was a direct interaction as determined by in vitro GST pull-down assays using recombinant GST-PRMT1 and recombinant human USP11 (Supplementary Fig. 1b). Together, these observations validate our mass spectrometry data. Given that some PRMT-interacting proteins are cofactors rather than substrates^35,36^, we conducted in vitro methylation assays and confirmed that GST-PRMT1 can directly methylate recombinant human USP11 (Fig. 1b). Given that PRMT1 is overexpressed^37^ and USP11 is dysregulated in breast cancer^38^, and that the MCF7 breast cancer cell line expresses high levels of PRMT1 and USP11 (Supplementary Fig. 1c), we also tested the ability of PRMT1 to methylate USP11 in this cell line. Using a modified in vitro methylation assay, MCF7 cellular proteins were first hypomethylated via the addition of the pan methyl-inhibitor AdOx, and USP11 was then isolated from cell lysates by immunoprecipitation. Methylation reactions were initiated via the addition of GST-PRMT1 and the methyl-donor SAM. Hypomethylated USP11 was more efficiently methylated by PRMT1 than USP11 isolated from cells not treated with AdOx, suggesting that in vivo, USP11 is a substrate for PRMT1 and that this can be revealed when PRMT1-methyl acceptor sites are made available through reduced in vivo USP11 methylation (Fig. 1c). To support this further, we determined the in vivo methylation status of USP11 by labelling 293T cells with radioactive [^3^H]-methionine, which serves as a precursor for the synthesis of [^3^H]-SAM. By blocking de novo protein synthesis via the addition of cycloheximide and chloramphenicol, any tritiated isotope incorporated into immunoprecipitated proteins is suggestive of protein methylation. Immunoprecipitation of Flag-USP11, but not the control Flag-GFP, demonstrated substantial isotope incorporation indicative of post-translational methylation (Fig. 1d). Importantly, we could detect methylation of endogenous USP11 in MCF7 cells, and that this methylation is significantly suppressed after knockdown of PRMT1 (Fig. 1e). Taken together, this data strongly suggests that USP11 is a bona fide PRMT1 substrate.

### PRMT1 methylates USP11 at R433

**Fig. 2:**
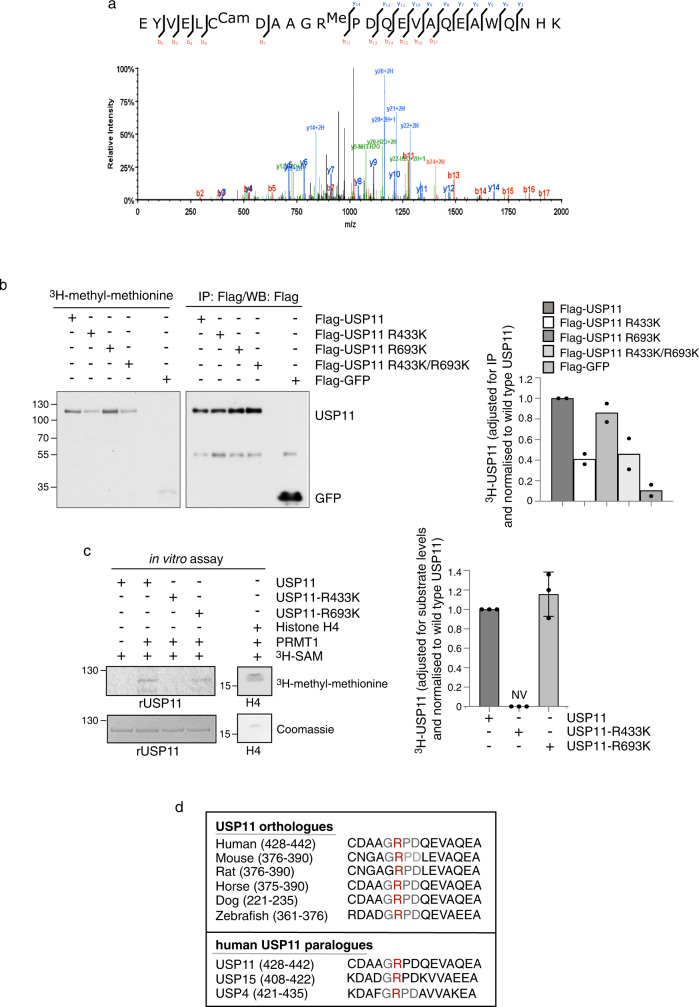
PRMT1 methylates USP11 at R433. **a** Higher energy Collisional Dissociation (HCD) MS/MS spectrum and the associated peptide sequence of the arginine methylated peptide EYVELCDAAGRPDQEVAQNHK, residues 423–443 derived from USP11. **b** R433 is a methyl-acceptor site in cells. Autoradiograph and immunoblots of 293T cells transfected with Flag-USP11, Flag-USP11-R433K, Flag-USP11-R693K and Flag-USP11-R433K/R693K, treated with CAM/CHX and labelled with [^3^H]-methyl-methionine. The graph represents the quantification of *n* = 2 independent experiments. Data are depicted as methyl incorporation adjusted for USP11 immunoprecipitation and normalised to wild-type USP11. **c** USP11-R433K is the major PRMT1-mediated methylation site as determined by in vitro methylation assay. Histone H4 serves as a positive control. The graph to the right represents the quantification of *n* = 3 independent experiments. Data are depicted as methyl incorporation adjusted for substrate levels (USP11) and normalised to wild-type USP11 (mean ± SD). NV no value. **d** Protein sequence alignment by MUSCLE (multiple sequence comparison by log-expectation) using USP11 as a reference, showing conservation of R433 in several USP11 orthologues and in USP11 paralogues, USP4 and USP15. Uncropped blots and processed graphical data provided as a Source Data file.

Although PRMT1 has been proposed to preferentially methylate RG/RGG motifs^21^, some substrates lack this consensus motif^25^ making in silico prediction of methyl-acceptor sites challenging. To determine the site of methylation on USP11, we analysed immunoprecipitated ectopically-expressed Flag- or HA-tagged USP11 by mass spectrometry. Six independent experiments revealed two putative methylated residues, R433 and R693 (Fig. 2a, Supplementary Fig. 1d and data not shown). However, in vivo analysis of USP11 methylation status in cells expressing either wild-type USP11, or USP11 where the target arginines had been mutated to lysines (R433K and R693K), validated R433 but not R693 as an in vivo methylation site (Fig. 2b). These findings were supported by in vitro methylation assays using recombinant PRMT1 incubated with either wild-type His-USP11, USP11-R433K or USP11-R693K recombinant protein (Fig. 2c). Indeed, the finding that the methylation of USP11 catalysed by PRMT1 was completely abolished by the R433K mutation strongly implies that at least in vitro, this is the sole residue targeted by PRMT1. This also suggests that the residual methylation detected in USP11-R433K expressing cells could be a consequence of additional, yet-to-be-identified, methylation sites catalysed by other PRMTs or lysine methyltransferases. Indeed, mass spectrometry studies have identified R241, K383 and R677 as being monomethylated^39^. Interestingly, R433 appears to be evolutionarily conserved within several species and within the USP11 paralogues USP4 and USP15 (Fig. 2d).

### Methylation of USP11 modulates its activity

**Fig. 3:**
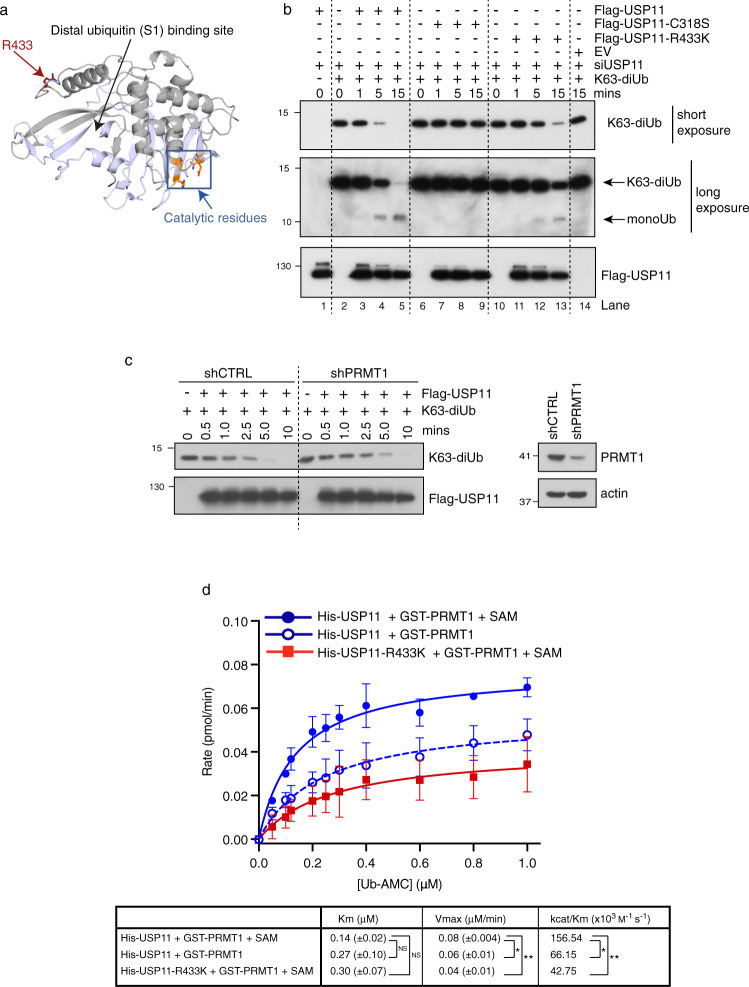
Methylation of USP11 at R433 regulates DUB activity. **a** Predicted structure of the catalytic core of USP11 using USP4 and USP15 structures as templates (PDB IDs: 2Y6E, 6GHA) showing the location of the distal (S1) ubiquitin-binding site, the catalytic residues (orange) and arginine 433 (red). **b** In vitro DUB assay using K63-linked di-ubiquitin (K63-diUb) as substrate. 293T cells were depleted of endogenous USP11 and then transfected with the indicated constructs and Flag-tagged USP11 immunoprecipitated. Following Flag peptide elution, immunoprecipitated USP11 was incubated with K63-diUb for time-course analysis of di-ubiquitin chain processing (representative image of *n* = 3 independent biological repeats). Lanes 1–14 were run on a single gel, dotted line is to aid interpretation. **c** Depletion of PRMT1 reduces USP11 activity as determined by in vitro DUB assay using K63-linked di-ubiquitin as substrate. Flag-USP11 was immunoprecipitated from 293T Flp-In-Flag-USP11 cells stably depleted for PRMT1 and peptide eluted. Flag-USP11 was incubated with K63-diUb for time-course analysis of di-ubiquitin chain processing (representative image of *n* = 3 independent biological repeats). All lanes were run on a single gel, the dotted line is to aid interpretation. **d** Methylation of USP11 alters its DUB activity. Recombinant wild-type or R433K His-tagged USP11 was incubated with GST-PRMT1, SAM (as indicated), and increasing concentrations of Ub-AMC. DUB activity was measured through fluorescence detection of cleaved AMC. Data were fitted according to Michaelis–Menten model using GraphPad Prism. Enzymatic parameters *K*_m_, *V*_max_ and catalytic efficiency (*k*_cat_/*K*_m_) are shown in the table below (mean ± SD; *n* = 3 independent biological repeats: one-way ANOVA and Tukey post hoc test: Vmax: His-USP11/GST-PRMT1/SAM versus His-USP11-R433K/GST-PRMT1/SAM **p = 0.0035; His-USP11/GST-PRMT1/SAM versus His-USP11/GST-PRMT1 *p = 0.0438; *k*_cat_/*K*_m_: His-USP11/GST-PRMT1/SAM versus His-USP11-R433K/GST-PRMT1/SAM **p = 0.0034; His-USP11/GST-PRMT1/SAM versus His-USP11/GST-PRMT1 *p = 0.0104. NS not significant. Uncropped blots and processed graphical data provided as a Source Data file.

Whilst the partially solved crystal structure of USP11 lacks information regarding the positioning of R433^40^, in silico comparative modelling of the catalytic core of USP11 based on available structures for the paralogues USP15 and USP4^41–43^ predicts that R433 is located on the surface, accessible for methylation by PRMT1, and close to the distal ubiquitin (S1) binding site (Fig. 3a). USP11 and USP4 can be regulated by a number of PTMs, including phosphorylation and auto-deubiquitylation^44–46^. Given the positioning of R433, we hypothesised that arginine methylation could influence the DUB activity of USP11. To investigate this, we determined the ex vivo catalytic activity of wild-type and methyl-deficient USP11. Here, 293T cells were depleted of endogenous USP11 and then transfected with siUSP11 resistant Flag-USP11, Flag-USP11-R433K, or catalytic inactive Flag-USP11-C318S^28^. USP11 proteins were then immunoprecipitated, peptide eluted, and incubated with K63-linked di-Ub chains, the preferred in vitro substrate for USP11^40^. Control immunoprecipitations derived from cells transfected with vector alone did not display any DUB activity, confirming that any associated activity was dependent on Flag-USP11 (Fig. 3b, lane 14). As expected, immunoprecipitation of Flag-USP11 robustly cleaved di-Ub to mono-Ub in a time-dependent manner (Fig. 3b lanes 2–5). In contrast, immunoprecipitated Flag-USP11-R433K decreased the hydrolysis of di-Ub-K63, although not as extensively as the catalytically inactive mutant, Flag-USP11-C318S (Fig. 3b, compare 5 min and 15 min time points). Similarly, depletion of PRMT1 in Flag-USP11 expressing cells resulted in a subtle decrease in USP11 DUB activity, as measured by ex vivo K63 di-Ub cleavage assay (Fig. 3c).

To support these findings and examine enzyme kinetics in more detail, we conducted an in vitro methylation-deubiquitylation coupled assay using the fluorogenic substrate ubiquitin-AMC (Ub-AMC) to measure USP11 enzymatic rates. Initially, recombinant wild-type His-USP11 or USP11-R433K was incubated with GST-PRMT1, with or without SAM. Given that commercially available asymmetric dimethyl antibodies are generally raised against RG/RGG motifs and hence do not cross-react with methylated USP11, we validated that our conditions were suitable for PRMT1 catalysis by performing the methylation reaction in parallel with recombinant histone H4 and confirmed in vitro methylation through immunoblot detection of asymmetric dimethylation at H4R3 (H4R3me2a) (Supplementary Fig. 1e). We then incubated the USP11 previously subjected to in vitro methylation with increasing concentrations of Ub-AMC and determined DUB activity through fluorescence detection of released AMC. The initial rate of the reactions was plotted against the Ub-AMC concentration and data fitted to a Michaelis–Menten model (Fig. 3d). Although the affinity of wild-type USP11 for substrate was not significantly changed after the methylation reaction (presumably generating methyl-USP11) compared to unmethylated USP11, as indicated by the *K*_m_, the catalytic efficiency was doubled in methyl-USP11-containing reactions (*k*_cat_/*K*_m_ = 156.54 × 10^3^ M^−1^ s^−1^) compared to non-methyl USP11 (*k*_cat_/*K*_m_ = 66.15 × 10^3^ M^−1^ s^−1^; *p* < 0.05; Fig. 3D). Moreover, this change in USP11 DUB activity was dependent on R433 as incubation of USP11-R433K with PRMT1 and SAM significantly reduced catalytic activity to that of unmethylated USP11 (USP11-R433K *k*_cat_/*K*_m_ = 42.75 × 10^3^ M^−1^ s^−1^; Fig. 3d). Importantly, the inability to methylate USP11 at R433 did not render USP11 catalytically inactive implying that methylation, although not critical, contributes to USP11 activity. Indeed, the finding that substrate affinity is comparable between unmethylated USP11 (*K*_m_ = 0.27) and USP11-R433K (*K*_m_ = 0.30) shows that the arginine to lysine substitution is not simply disrupting structural elements associated with catalysis. Taken together, our in vitro and in vivo data suggest that whilst methylation at R433 of USP11 is not essential, it may regulate USP11 catalytic activity, possibly functioning as a fine-tuning mechanism.

### Methylation of USP11 at R433 affects HR repair

**Fig. 4:**
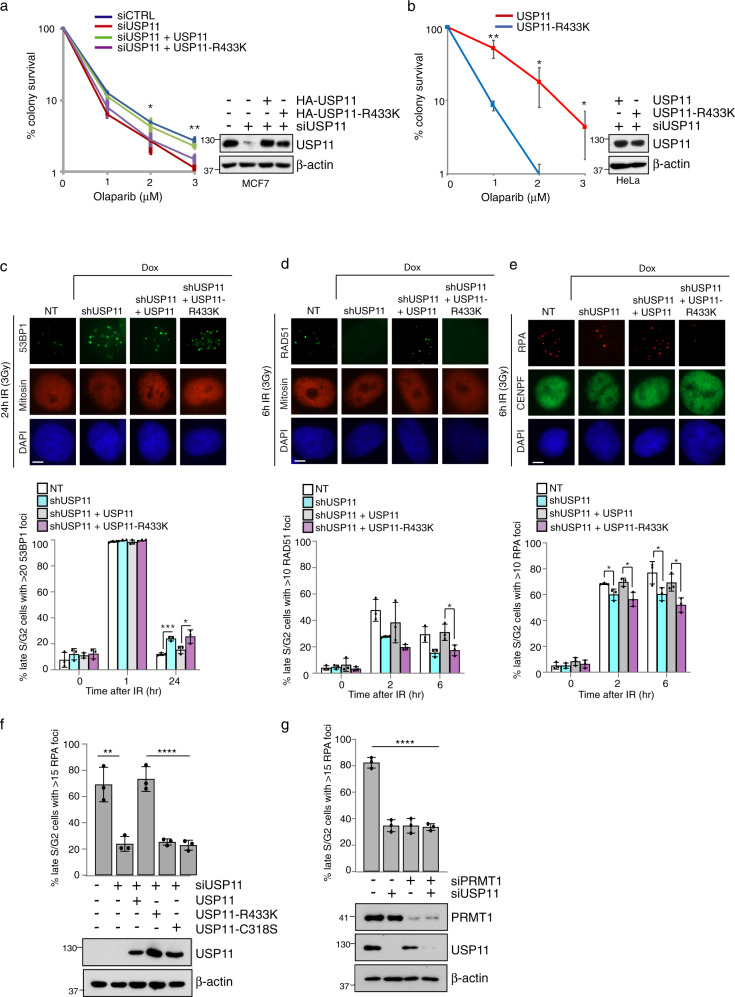
Methylation of USP11 at R433 is required for homologous recombination-mediated repair of DSBs. **a** MCF7 cells stably expressing USP11 or USP11-R433K were transfected with siUSP11, exposed to increasing doses of olaparib and cell viability determined by colony survival assay (mean ± SD; *n* = 3 independent biological repeats; Student’s *t* test (two-sided, equal variance) *p = 0.010, **p = 0.00257 comparing USP11 with USP11-R433K). **b** HeLa-USP11 and HeLa-USP11-R433K cells were transfected with siUSP11, exposed to increasing doses of olaparib and cell viability determined by colony survival assay (mean ± SD; *n* = 3 independent biological repeats; Student’s *t* test (two-sided, equal variance) **p = 0.006 (1 μM); *p = 0.0412 (2 μM); *p = 0.0539 (3 μM)). **c**–**e** MCF7 cells stably expressing doxycycline-inducible shUSP11 and ectopic USP11 or USP11-R433K were treated with doxycycline for 72 hrs, exposed to 3 Gy IR, and harvested at the time points indicated (mean ± SD; *n* = 3 independent biological repeats). Mitosin or CENPF-positive cells were scored for: **c** 53BP1 (Student’s *t*-test (two-sided, equal variance): *p = 0.04, ***p = 0.0005); **d** RAD51 (Student’s *t* test (two-sided, equal variance): *p = 0.02 NT versus shUSP11; *p = 0.04 shUSP11/USP11 versus shUSP11/USP11-R433K); **e** RPA (Student’s *t* test (two-sided, equal variance): *p = 0.03 NT versus shUSP11 (2 h post IR); *p = 0.02 shUSP11/USP11 versus shUSP11/USP11-R433K (2 h post IR); *p* = 0.05 NT versus shUSP11 (6 h post IR); *p = 0.03 shUSP11/USP11 versus shUSP11/USP11-R433K (6 h post IR)). Scale bar represents 5 μm. **f** HeLa cells stably expressing USP11, USP11-R433K or USP11-C318S were transfected with siUSP11, exposed to 3 Gy IR, and harvested 6 hrs later. CENPF-positive cells were scored for RPA foci formation (mean ± SD; *n* = 3 independent biological repeats, **p = 0.0057 (Student’s *t* test (two-sided, equal variance); ****p < 0.0001; (ordinary one-way ANOVA and Tukey post hoc test)). **g** PRMT1 and USP11 are epistatic in the regulation of RPA foci formation after IR (3 Gy) in HeLa late S/G2 cells (mean ± SD; *n* = 3 independent biological repeats; ****p < 0.0001; one-way ANOVA and Tukey post hoc test). Uncropped blots, raw and processed graphical data provided as a Source Data file.

Several lines of evidence have implicated USP11 and PRMT1 within the DDR, particularly as important components of HR-mediated repair^13,14,17,27–29^. Given that a mechanistic link between these two enzymes has not been studied, we generated a reconstitution system whereby MCF7 and HeLa cells were engineered to stably express ectopic HA-tagged USP11 or USP11-R433K in the cellular context of endogenous USP11 depletion. Using colony survival assays, we confirmed previous findings from U-2-OS^28^ and 293T cells^27^, that depletion of USP11 in MCF7 cells sensitises cells to olaparib (Fig. 4a), a genotoxic agent that produces lesions that require HR for repair^47^. More interestingly, whilst expression of USP11 rescued the defect in DNA repair induced by USP11 depletion, confirming the specificity of our siRNA sequences, expression of USP11-R433K could not, implying that methylation is required for USP11 to effectively function in the repair of olaparib-induced lesions (Fig. 4a, b). Supporting this, doxycycline-inducible knockdown of USP11 and re-expression of methyl-deficient USP11 resulted in impaired clearance of 53BP1 foci in late S/G2 cells indicating persistent and unrepaired DSBs (Fig. 4c and Supplementary Fig. 2a). These findings clearly show that USP11 methylation plays a fundamental regulatory role during DSB repair.

**Fig. 5:**
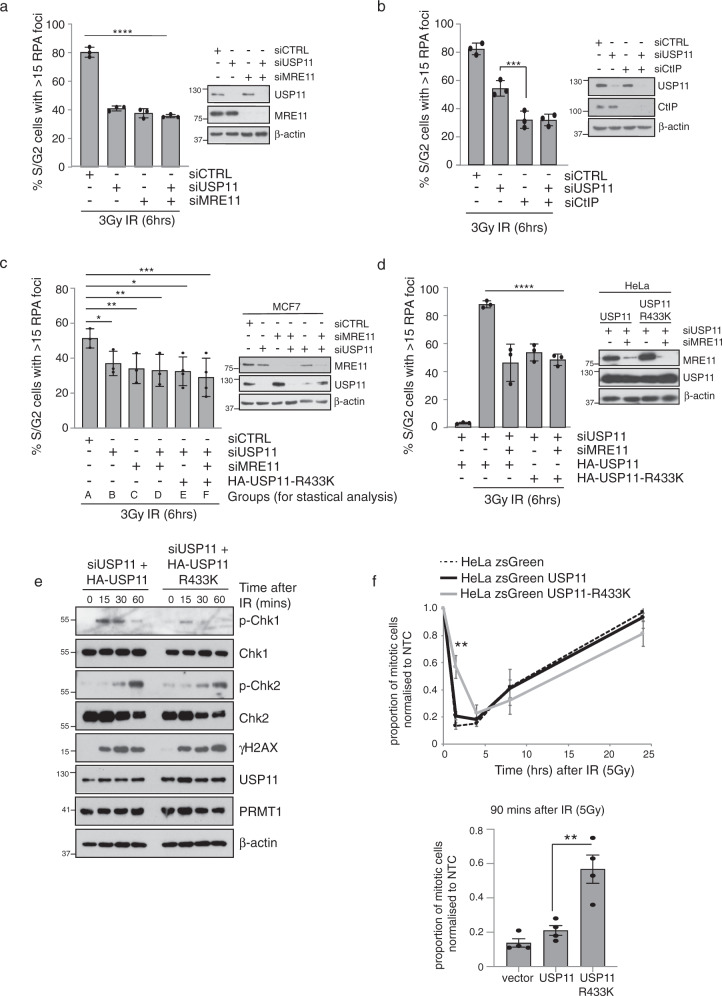
Methyl-USP11 regulates end-resection, ATR signalling and G2/M checkpoint. **a** USP11 and MRE11 are epistatic in the regulation of RPA foci formation after IR (3 Gy) in late S/G2 HeLa cells. CENPF-positive cells were scored for RPA foci formation (mean ± SD; *n* = 3 independent biological repeats; ****p < 0.0001; one-way ANOVA and Tukey post hoc test). **b** USP11 is not epistatic with CtIP in the regulation of RPA foci formation after IR (3 Gy) in late S/G2 HeLa cells (mean ± SD; *n* = 3 independent biological repeats; Student’s *t* test (two-sided, equal variance): ***p = 0.0096). **c** Methylated USP11 is epistatic with MRE11 in the regulation of RPA foci formation after IR in late S/G2 MCF7 cells. CENPF-positive cells were scored for RPA foci formation (mean ± SD; *n* = 3 independent biological repeats; one-way ANOVA and Tukey post hoc test: groups A and B *p = 0.031; groups A–C **p = 0.0046; groups A–D **p = 0.0022; groups A–E *p = 0.0162; groups A–F ***p = 0.0006). **d** Methylated USP11 is epistatic with MRE11 in the regulation of RPA foci formation after IR in late S/G2 HeLa cells. CENPF-positive cells were scored for RPA foci formation (mean ± SD; *n* = 3 independent biological repeats; one-way ANOVA and Tukey post hoc test: ****p < 0.0001). **e** HeLa-USP11-R433K cells display defective ATR signalling. HeLa-USP11 or HeLa-USP11-R433K cells were transfected with siUSP11, exposed to 10 Gy IR and then harvested at the time-points indicated for immunoblot analysis. Representative image of *n* = 3 independent biological repeats. **f** HeLa-USP11-R433K cells exhibit a G2/M checkpoint deficiency. Cells were treated with 5 Gy IR and harvested at the time points indicated. Proportion of mitotic cells was determined by phospho-Histone H3 (S10) staining (mean ± SEM; *n* = 3 biological repeats; Student’s *t* test (two-sided, equal variance): **p = 0.0023). Data on bar graph underneath represents the 90 min time point (mean ± SEM; *n* = 3 biological repeats; Student’s *t* test (two-sided, equal variance): **p = 0.0023). Uncropped blots, raw and processed graphical data provided as a Source Data file.

USP11 positively regulates the BRCA1–PALB2–BRCA2 complex enabling RAD51 loading onto ssDNA and homology-mediated strand invasion^27^. During this process, the ubiquitylation of PALB2 by KEAP1, which blocks its interaction with BRCA1, suppresses BRCA2 recruitment to DNA damage sites during the G1 phase of the cell cycle. During S/G2, USP11 expression is upregulated, counteracting KEAP1-dependent ubiquitylation of PALB2, enabling a reversal of complex inhibition and effective HR repair in the correct stage of the cell cycle^27^. In line with this study, we found that depletion of USP11-reduced RAD51 foci formation in the late S/G2 phase in both MCF7 and HeLa cells after ionising radiation, which could be rescued by re-expression of wild-type USP11 (Fig. 4d and Supplementary Fig. 2b). Interestingly, we found that, unlike wild-type USP11, methyl-deficient USP11 (R433K) expressing cells still exhibited defective RAD51 foci formation (Fig. 4d and Supplementary Fig. 2b). Although this implies that methyl-USP11 regulates RAD51 loading, we found that events upstream of RAD51 nucleofilament formation were also dependent on USP11 methylation, including RPA foci formation in late S/G2 cells indicative of a DNA resection defect (Fig. 4e, f). Indeed, the importance of USP11 methylation is underscored by our observation that the extent of defective RPA loading was similar to that observed in cells expressing catalytically inactive USP11 (USP11-C318S) (Fig. 4f) and that PRMT1 and USP11 functioned in an epistatic manner with respect to promoting the recruitment of RPA to DSBs (Fig. 4g). Importantly, our findings are not due to alterations in the cell cycle, as in agreement with others^29^, knockdown of USP11 has no effect on cell cycle progression (Supplementary Fig. 2c). Finally, in comparison to USP11-C318S, the expression of USP11-R433K did not affect PALB2 deubiquitylation (Supplementary Fig. 2d). Taken together, our data indicate that USP11 methylation is not required for the very early stages of DSB repair sensing leading to ATM activation, as measured by Chk2 phosphorylation (Fig. 5e), and 53BP1 recruitment (Fig. 4c) but highlight a role for it during DNA end-resection that is independent of PALB2 deubiquitylation. Hence, the methylation of USP11 appears to direct USP11 activity towards a particular stage of HR repair, namely DNA end-resection and RPA loading, rather than the formation of the BRCA1–PABL2–BRCA2 complex.

### Methylation of USP11 regulates MRE11 activity during HR repair

DNA end-resection is a critical step that initiates and commits a DSB to repair by HR. In eukaryotic cells, the endonuclease activity of MRE11 nicks the DNA within 300 bp of the DSB, and then, through the use of its 3′–5′ exonuclease activity, extends the formation of ssDNA towards the break end^48^. This “short-range” resection primes other “long-range” exonucleases, such as EXO1 and DNA2, that possess 5′–3′ nuclease activity enabling the formation of long 3′ ssDNA tails that are rapidly coated with RPA^49^. A second short-range nuclease, CtIP, stimulates MRE11 activity when phosphorylated by CDKs. Importantly, the CDKs that phosphorylate CtIP are specific to the S/G2 phase of the cell cycle, thereby ensuring that initiation of end-resection is maximal when a sister chromatid is present^50,51^. Interestingly, we found that co-depletion of USP11 with MRE11 in MCF7 and HeLa cells did not further reduce RPA foci formation compared to individual knockdowns, suggesting that these enzymes are functioning in the same pathway (Fig. 5a, c and Supplementary Fig. 2e). In contrast, RPA foci formation after CtIP knockdown was not epistatic with USP11 knockdown (Fig. 5b and Supplementary Fig. 2f). Interestingly, we found that reconstitution of USP11 depleted cells with methyl-deficient USP11 was also epistatic with MRE11 depletion in MCF7 and HeLa cells (Fig. 5c, d), thereby functionally linking USP11 methylation to MRE11. Supporting this, USP11-R433K reconstituted cells displayed reduced Chk1 phosphorylation compared to wild-type USP11 reconstituted cells (Fig. 5e). Likewise, ectopic over-expression of USP11-R433K resulted in a 2.7-fold increase in cells entering mitosis 90 min after DNA damage compared to empty vector and USP11 wild-type expressing cells (Fig. 5f). This is consistent with a role for the MRN complex facilitating ATR activation through the formation of RPA-coated ssDNA and G2/M checkpoint activation after DSB induction^20,52^. Hence, phenotypes of methyl-deficient USP11 appear to align with those of defective MRE11 activity.

### PRMT1-USP11 crosstalk regulates MRE11 activity

USP11 methyl-deficient cells have a G2/M checkpoint defect similar to that observed in MEFs derived from the *Mre11*^*RR/KK*^ transgenic mouse^20^. This, coupled with the epistatic nature of MRE11 and methyl-deficient USP11 (Fig. 5c, d), implies that methylation of USP11 regulates MRE11, possibly through promoting MRE11 methylation. Recently, GFI1 was shown to directly interact with PRMT1 and function as a PRMT1 cofactor facilitating MRE11 methylation^18^. Given that GFI1 is expressed within T-cells and not within our epithelial HeLa/MCF7 cell lines (Supplementary Fig. 3a) we hypothesise that methylated USP11 could be functioning in a manner analogous to GFI1 by regulating PRMT1 activity. Whilst we saw no global change in asymmetric dimethylarginine (ADMA) levels after USP11 knockdown, or after expression of wild-type or catalytically inactive USP11 (Supplementary Fig. 3b), we rationalised that as the majority of proteins that are detected by pan-ADMA antibodies are highly abundant splicing factors, such an approach may not reveal subtle changes in PRMT1 activity. We therefore decided to take an in vitro approach to understand how USP11 could be affecting in vivo PRMT1 activity. Here, we transfected HeLa cells with Flag-PRMT1, manipulated USP11 levels by siRNA or overexpression, immunoprecipitated Flag-PRMT1 and purified the enzyme by peptide elution. We then assessed the activity of PRMT1 by incubating with recombinant histone H4 and detecting asymmetric dimethylation at histone H4 at R3 (H4R3me2a) as an indicator of in vivo PRMT1 activity. Using this ex vivo approach, we found that depletion of USP11 reduced H4R3me2a levels (Supplementary Fig. 3c), whilst overexpression increased H4R3me2a (Supplementary Fig. 3d). Next, we wanted to determine whether methylation and/or the catalytic activity of USP11 influences PRMT1 activity. Again, we depleted USP11 from cells and reconstituted with wild-type USP11, USP11-R433K or USP11-C318S, and then immunoprecipitated PRMT1 to determine activity ex vivo. Interestingly, methylation of USP11 and its catalytic activity, although not essential, did contribute to PRMT1 activity (Supplementary Fig. 3e).

**Fig. 6:**
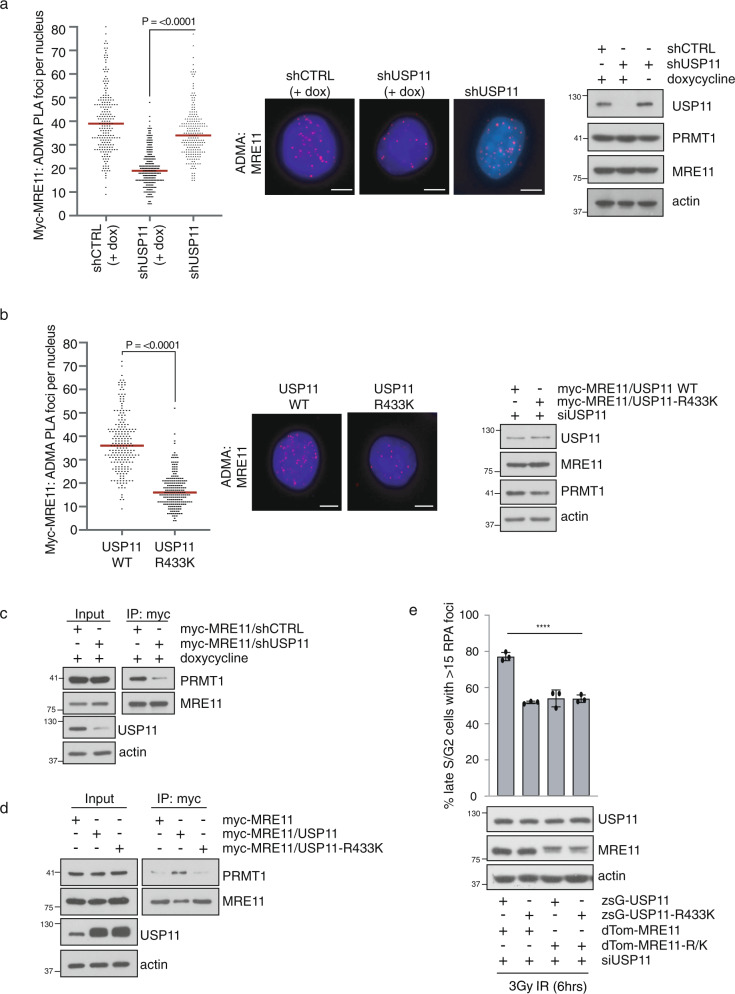
Methylated USP11 regulates PRMT1 activity towards MRE11. **a** USP11 regulates MRE11 methylation. Knockdown of USP11 was induced by doxycycline treatment of HeLa-pTRIPZ-shCTRL and shUSP11 cells, and asymmetric dimethylation of MRE11 was detected by PLA (antibodies directed towards myc-tagged MRE11 and ADMA). Data are from three independent biological experiments (median PLA foci for shUSP11 (+dox) and shUSP11 (−dox) were 19 and 34, respectively; Mann–Whitney *n*_1_ = 203; *n*_2_ = 207; *p* < 0.0001; two-tailed). The middle and right panels display representative PLA images and immunoblots. Scale bar represents 5 μm. **b** Methyl-USP11 regulates MRE11 methylation. HeLa cells stably expressing myc-MRE11 and USP11 wildtype or R433K were transfected with siUSP11 to deplete endogenous protein, and levels of MRE11 methylation were detected by PLA. Data are from three independent experiments (median PLA foci for USP11 WT and USP11-R433K were 36 and 16, respectively; Mann–Whitney *n*_1_ = 209; *n*_2_ = 207; *p* < 0.0001; two-tailed). The middle and right panels display representative PLA images and immunoblots. Scale bar represents 5 μm. **c** Depletion of USP11 reduced PRMT1 and MRE11 interactions as determined by co-immunoprecipitation. HeLa-myc-MRE11/pTRIPZ-shCTRL or HeLa-myc-MRE11/pTRIPZ-shUSP11 cells were treated with doxycycline and MRE11 immunoprecipitated with anti-myc antibodies. Associated PRMT1 was detected by immunoblotting (representative image of *n* = 3 independent biological experiments). **d** Expression of USP11-R433K decreases PRMT1 and MRE11 interactions in HeLa cells as determined by co-immunoprecipitation. HeLa-myc-MRE11 cells were generated to stably express USP11 or USP11-R433K, and MRE11 was immunoprecipitated with anti-myc antibodies. Associated PRMT1 was detected by immunoblotting (representative image of *n* = 3 independent biological experiments). **e** PRMT1-mediated methylation of USP11 and MRE11 is epistatic for RPA foci formation after ionising radiation in late S/G2 cells. HeLa cells stably expressing USP11, USP11-R433K, MRE11 and MRE11-R/K (MRE11 mutated in all PRMT1-methyl acceptor sites) were transfected with siUSP11, exposed to 3 Gy IR, and harvested 6 h later. CENPF-positive cells were scored for RPA foci formation (mean ± SD; *n* = 3 independent biological experiments; one-way ANOVA and Tukey post hoc test: ****p < 0.0001). Uncropped blots, raw and processed graphical data provided as a Source Data file.

To further support our hypothesis that the changes in PRMT1 activity induced by methyl-USP11 are directed towards MRE11, we analysed MRE11 methylation status through two independent approaches: proximal ligation assay (PLA) and MRE11 immunoprecipitation followed by ADMA immunoblotting. PLA was conducted by generating HeLa myc-tagged MRE11 cells, and MRE11 methylation was detected by myc-9E10/Asym26 antibody pairing because the pan-Asym26 antibody is known to cross-react with methyl-MRE11^18^. Validation of the specificity of our methyl-MRE11 PLA signal was confirmed by depleting PRMT1 and expression of MRE11 mutated at methylated arginines within the GAR domain (MRE11 R/K)^20^ (Supplementary Fig. 4a, b). Residual PLA signal detected in MRE11-R/K cells is most likely due to dimer formation with endogenous wild-type protein^53^. We found that depletion of USP11 significantly decreased MRE11 methylation (Fig. 6a and Supplementary Fig. 4c). More notably, methylation at R433 of USP11 appears to be important for this event as reconstitution of USP11 knockdown cells with USP11-R433K resulted in reduced methyl-MRE11 levels compared to cells expressing wild-type USP11 (Fig. 6b and Supplementary Fig. 4d). Critically, depletion of USP11 or expression of USP11-R433K did not affect total MRE11 levels. Finally, depletion of endogenous USP11 in stable MCF7-USP11 wildtype and R433K cell lines, followed by immunoprecipitation of MRE11 detected a small change in endogenous MRE11 methylation (Supplementary Fig. 4e). Mechanistically, knockdown of USP11 did not affect PRMT1 homodimer formation (Supplementary Fig. 4f), an event that facilitates SAM binding and catalytic activity^54^, but did regulate MRE11-PRMT1 interaction (Fig. 6c). Interestingly, this was again dependent on USP11 methylation status as overexpression of wild-type USP11 increased PRMT1-MRE11 interactions, whilst USP11-R433K did not (Fig. 6d). Finally, this interplay between methyl-USP11 and methyl-MRE11 was functionally relevant because stable co-expression of USP11-R433K and MRE11-R/K resulted in an epistatic interaction regarding RPA foci formation after IR-induced DSBs (Fig. 6e). Taken together, our data implies that the methylation of USP11 at R433 facilities PRMT1/MRE11 interactions that lead to enhanced MRE11 methylation. Hence, PRMT1-mediated methylation of both USP11 and MRE11 is required for effective DNA end-resection during HR-mediated repair, and these two arginine methylation events are functionally linked.

### USP11 deubiquitylation of PRMT1 regulates methyltransferase activity

**Fig. 7:**
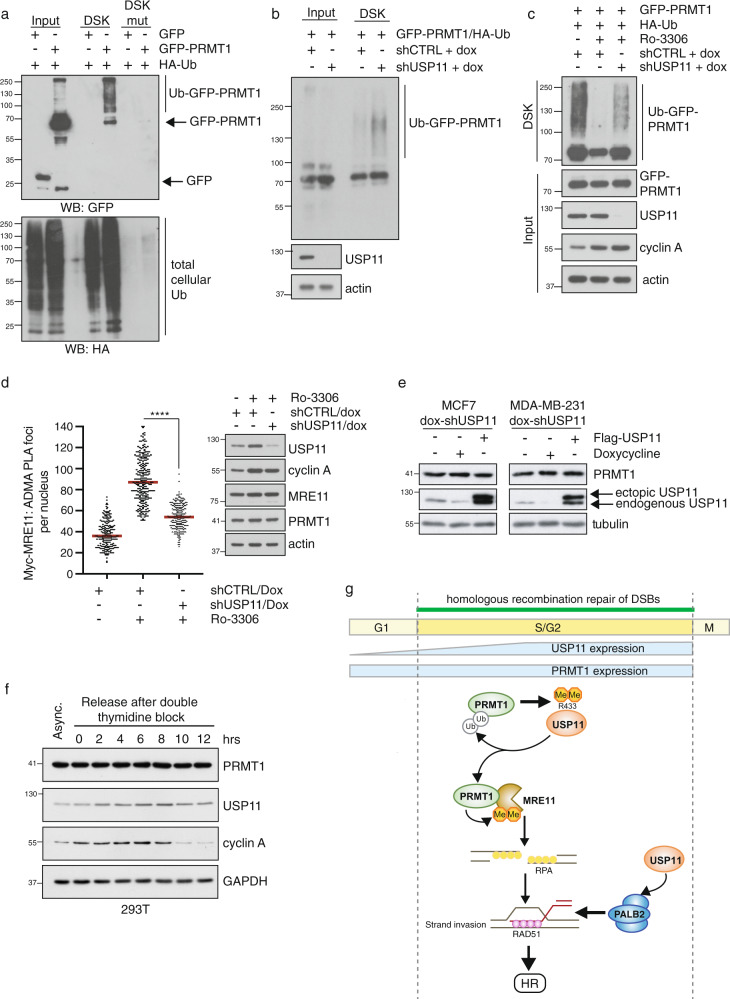
USP11 deubiquitylates PRMT1, increasing MRE11 methylation. **a** PRMT1 is ubiquitylated in 293T cells as determined by MultiDSK pulldown. DSK mut = MultiDSK that cannot bind ubiquitin (representative image of *n* = 3 independent biological experiments). **b** PRMT1 ubiquitylation status is USP11-dependent. 293T-pTRIPZ-shCTRL or shUSP11 cells were transfected with the indicated constructs and treated with doxycycline for 48 hrs. PRMT1 ubiquitylation was determined by MultiDSK pulldown (representative image of *n* = 3 independent experiments). **c** PRMT1 ubiquitylation is decreased in G2/M arrested cells in a USP11-dependent manner. HeLa-pTRIPZ-shUSP11 cells were transfected with the indicated constructs and doxycycline was added for 72 hrs. Ro-3306 (9 μM) was added 20 hrs prior to lysis and ubiquitylation status was determined by MultiDSK pulldown (representative image of *n* = 3 independent experiments). **d** MRE11 methylation is increased in G2/M arrested cells in a USP11 dependent manner. HeLa myc-MRE11/pTRIPZ-shCTRL or shUSP11 cells were treated with doxycycline for 48 h and then Ro-3306 (9 μM) for a further 20 hrs. Levels of MRE11 methylation were determined by myc-ADMA PLA. Data are from three independent biological experiments, with >75 nuclei scored per repeat (medium PLA foci for shCTRL (+Ro-3306) and shUSP11 (+Ro-3306) were 87 and 54, respectively; Mann–Whitney *n*_1_ = 243; *n*_2_ = 234; *p* < 0.0001; two-tailed). **e** Stable knockdown or overexpression of USP11 does not change PRMT1 protein levels in MCF7 and MDA-MB-231 cells. Representative image of *n* = 3 independent experiments. **f** USP11 but not PRMT1 expression levels are upregulated in S/G2 cells. 293T cells were synchronised by double thymidine block, released into complete media and harvested at the time points indicated. Cyclin A levels indicate cells in the S/G2 phase (representative image of *n* = 2 independent biological experiments). **g** Proposed model of cross-talk between arginine methylation, deubiquitylation and the control of DNA end-resection. USP11 levels increase in the S/G2 phase of the cell cycle providing substrate for PRMT1 to methylate USP11 at R433. In turn, methyl-USP11 catalyses the deubiquitylation of PRMT1, enhancing PRMT1-MRE11 interactions and promoting MRE11 methylation in S/G2 cells. This stimulates DNA end-resection that commits a cell to HR-mediated repair of DSBs. Unmethylated USP11 deubiquitylates PALB2. Uncropped blots and raw graphical data provided as a Source Data file.

To date, very few cellular substrates for USP11 have been identified. Given our finding that USP11 regulates PRMT1 activity, we hypothesised that PRMT1 may be a direct substrate of USP11. PRMT1 has previously been shown to be ubiquitylated by TRIM48 and Fbxl17^55,56^. Indeed, we show that PRMT1 is a highly ubiquitylated protein through His-tag ubiquitin pull-down (Supplementary Fig. 5a). Likewise, incubation of cell lysates with MultiDsk (DSK), a recombinant ubiquitin-binding protein that comprises of five repeats of yeast Dsk2 UBA domains fused to GST^57^, affinity-purified ubiquitylated PRMT1, whilst incubation with MultiDsk mutated in the UBA domain (DSK-mut) did not (Fig. 7a). More interestingly, we did indeed identify USP11 as a PRMT1 DUB because knockdown of USP11 increased PRMT1 ubiquitylation, whilst ectopic overexpression suppressed ubiquitylation in a USP11 catalytic-dependent manner (Fig. 7b and Supplementary Fig. 5b). USP11-mediated deubiquitylation of PRMT1 appears to be specific as knockdown of USP11 does not affect MRE11 ubiquitylation (Supplementary Fig. 5c). USP11 levels are elevated in S/G2 cells (Fig. 7d, f and ref. ^27^), therefore to further support PRMT1 as a USP11 substrate, we wondered if USP11-mediated deubiquitylation of PRMT1 was enhanced in this stage of the cell cycle. Indeed, treatment of cells with the CDK1 inhibitor Ro-3306 that arrests cells in G2/M (Supplementary Fig. 5d) leads to a reduction of PRMT1 ubiquitylation that was partially USP11 dependent (Fig. 7c). Significantly, these changes in PRMT1 ubiquitylation also corresponded to changes in methyl-MRE11, such that G2/M arrest increases MRE11 methylation in a USP11 dependent manner (Fig. 7d), thereby further linking USP11, PRMT1 and MRE11.

Ubiquitylation has been suggested to regulate PRMT1 stability^55,56^, however, the fact that we could easily detect PRMT1 ubiquitylation in the absence of proteasomal inhibition is strongly suggestive that a proportion of ubiquitylated PRMT1 is not targeted for degradation. Supporting this, the addition of MG132 only marginally increased PRMT1 ubiquitylation (Supplementary Fig. 5a), whilst protein levels of PRMT1 in two different breast cancer cell lines were unchanged after doxycycline-induced depletion or overexpression of USP11 (Fig. 7e). Interestingly, ectopic expression of USP11 stabilises endogenous USP11 levels (Fig. 7e). Since USP11 is known to auto-deubiquitylate^44^, it is plausible that ectopic USP11 could deubiquitylate endogenous USP11 leading to increased stability. Further evidence that USP11 is not targeting degradative ubiquitin chains on PRMT1 is provided by our findings that the cell cycle-associated increase in USP11 levels was not accompanied by changes in PRMT1 expression (Fig. 7f). These observations support the notion that USP11 is removing PRMT1-ubiquitin chains other that are not K48 or K11 linked. Indeed, in vitro, USP11 showed a preference for processing K63 di-Ub chains^40^. Furthermore, loss of USP11-dependent PALB2 deubiquitylation did not affect PALB2 protein levels^27^.

Thus, our data demonstrate that PRMT1 is targeted for deubiquitylation by USP11, which serves to fine-tune the enzymatic activity of PRMT1 enabling targeted methylation during a specific cellular process.

## Discussion

Crosstalk and integration of distinct PTMs is critical for an effective and controlled DDR and has been extensively studied in the context of phosphorylation and ubiquitylation. In this study, we have identified a complex interplay between the DUB enzyme USP11 and arginine methyltransferase PRMT1. We identify USP11 as a previously uncharacterised PRMT1 substrate, and show that methylation at R433, although not essential, appears to contribute to USP11 DUB activity. Correspondingly, we have also demonstrated that PRMT1 is a ubiquitylated protein which is targeted for deubiquitylation by USP11, and that methylated USP11 promotes PRMT1 activity. According to our model (Fig. 7g), this identified USP11-PRMT1 axis could be important for ensuring tight regulation of DNA damage repair mediated by HR because PRMT1-mediated methylation of USP11 at R433 is necessary for proper DNA end-resection induced by PRMT1-mediated methylation of MRE11. Once DNA end-resection has occurred, unmethylated USP11 acts on additional steps of the HR pathway, including the formation of the BRCA1–PALB2–BRCA2 complex that enables RAD51-driven homology strand invasion and D-loop formation^27^. Taken together, our findings identify DNA end-resection and RPA loading as a previously uncharacterised role for USP11 during HR-mediated repair of DSBs, and that arginine methylation confers a mechanism that enables separation of USP11 functions, specifying enzymatic activity.

A key observation in this study is that PRMT1-mediated USP11 methylation is biochemically and functionally linked to PRMT1-dependent MRE11 methylation, implying the existence of a feed-forward regulatory mechanism connecting arginine methylation and deubiquitylation. Supporting this, USP11-R433K reduces PRMT1/MRE11 interactions and MRE11 methylation, and cells expressing methyl-deficient USP11 are defective in Chk1 activation and G2/M DNA damage checkpoint control, phenotypes exhibited by mutation of the MRE11 GAR motif targeted by PRMT1^14,20^. Moreover, co-expression of MRE11-R/K and USP11-R433K reduces RPA foci formation to a similar extent as expression of either alone, implying functional epistasis. Enzymatic feed-forward loops can rapidly amplify signals but require tight regulation to prevent uncontrolled activation. We, and others, have demonstrated that USP11 protein levels increase during the S/G2 cell cycle phase^27^. Given that PRMT1 levels remain constant, we speculate that USP11 is the principal initiator of this feed-forward cycle. In this scenario, USP11 levels are elevated in S/G2 cells which leads to an accumulation of substrate for PRMT1 and a localised increase in methyl-USP11. Methylation increases USP11 enzymatic activity leading to the deubiquitylation of at least one substrate, PRMT1. Although we were unable to detect an increase in ectopically expressed USP11 methylation after DNA damage or in G2/M arrested cells using in vivo ^3^H-methionine labelling of cells (Supplementary Fig. 5e), we cannot rule out that a small proportion of USP11 is differentially methylated but below the threshold of detection via this approach. Methylated USP11 then promotes PRMT1 activity towards MRE11 that enables DNA end-resection and RPA loading (Fig. 7g). Hence, USP11 is functioning as both a substrate and cofactor of PRMT1. Our evidence implies that neither USP11 methylation nor PRMT1 deubiquitylation is essential for their respective activities, suggesting that these PTMs function as a fine-tuning mechanism in response to DSBs, or that a restricted sub-pool of the enzyme is dynamically modified and the effect is diluted within the whole protein population. Regardless, our findings help explain how enzymes that are known to have multiple substrates, even within a single pathway, can control specific events.

Excessive MRE11-dependent resection is detrimental to genome integrity, hence mechanisms to control PRMT1 methylation of MRE11 must exist to prevent aberrant exonuclease activity. In T-cells, GFI1 acts as an adaptor protein facilitating PRMT1/MRE11 interactions, thereby promoting MRE11 methylation^18^. Since GFI1 is a lymphoid specific protein, we propose that methyl-USP11 acts as an alternative mechanism for the regulation of MRE11 methylation. Vadnais et al.^18^, also suggested that GFI1 regulates PRMT1 activity towards a second substrate, 53BP1. However, we did not identify any role for USP11 or methyl-USP11 in the activation of ATM and the recruitment of 53BP1 to DSBs. Together, our studies add to the growing awareness that whilst PRMT1 methylates 53BP1 it is unlikely to contribute to 53BP1 DNA repair function^14,18,58^.

Whilst the majority of studies examining PRMT1 have focused on substrate identification, very few have determined how PRMT1 activity is directed to specific substrates. Although the mechanism by which PRMT1 deubiquitylation promotes methyltransferase activity is currently unknown, we can rule out increased PRMT1 protein stability as depletion or expression of catalytic inactive/methyl-deficient USP11 did not alter expression levels. To date, mass spectrometry approaches have identified at least 13 ubiquitylated lysine residues on PRMT1^39^, the majority of which are surface-facing and thus accessible for modification. Of interest, K313 (nomenclature to human PRMT1 isoform 1) is C-terminal adjacent to the ^310^THW loop, a motif important for catalytic activity. Indeed, phosphorylation of Y309 has been proposed to disrupt substrate binding and catalysis. Since K313 lies adjacent to Y309, one would predict that ubiquitylation of K313 could also severely disrupt activity. Another interesting putative site of ubiquitylation that has been detected in numerous proteomic approaches is K233, located at the base of the dimer arm, a region that is important for PRMT1 catalytic activity^54^. Although ubiquitylation could potentially sterically disrupt dimerisation (Supplementary Fig. 6a), we found that depletion of USP11 did not alter PRMT1 dimer formation. Alternatively, we found that USP11 regulated PRMT1/MRE11 interaction, implying that deubiquitylation of PRMT1 facilitates substrate binding. Unfortunately, biochemical mapping of ubiquitylation sites is notoriously difficult, either because the ubiquitin “jumps” to adjacent lysine residues after targeted mutation, or the proportion of ubiquitylated protein at the target site is a minor species, so is undetected amongst other ubiquitylation events. Indeed, expression of either PRMT1-K128/K131/K134R, PRMT1-K228/K233/K342R or PRMT1-K313R failed to suppress USP11-mediated deubiquitylation of PRMT1 (Supplementary Fig. 6b, c). Future quantitative di-Gly mass spectrometry approaches and the identification of site(s) on PRMT1 targeted by USP11 after DSB induction in conjunction with in silico modelling should lead to further mechanistic insight.

In a similar manner, little is known about how DUBs direct substrate specificity. Generally, USP family members are promiscuous in the type of ubiquitin chain editing, with protein-protein interactions and/or PTM contributing to substrate selectivity^59^. Recently, USP11 was shown to be phosphorylated at S453 by S6 kinase driving USP11-eIF4B interactions and the deubiquitylation of eIF4B^46^. Hence, in a similar manner to S453, R433 methylation could be modulating substrate binding or altering complex formation. In line with this, USP11 co-purifies with the NuRD chromatin remodelling complex promoting H2A/H2B deubiquitylation and timely disassembly of DNA repair factors^29^. It would be interesting to determine if the USP11 interactome is altered during the initial end-resection stages of HR repair and whether this is dependent on R433 methylation.

Breast cancer patients expressing high levels of PRMT1 but low levels of USP11 are associated with a poor survival rate (Supplementary Fig. 7), which could be a consequence of defective end-resection thereby favouring error-prone NHEJ and genome instability. Supporting this, interrogation of the COSMIC database has revealed that USP11-R433 and the equivalent site in its most closely related family members USP15 and USP4 are mutated in endometrium, lung and AML cancers respectively^60^. Somewhat paradoxically, many chemotherapies function by perturbing DNA repair mechanisms, with the principal aim of promoting catastrophic DNA damage that is incompatible with cellular survival. PRMT1 and USP11 inhibitors are in preclinical development^61,62^, hence drug targeting this enzyme axis may provide a route to increase the effectiveness of DNA damaging chemotherapeutic agents that require HR for repair.

## Methods

### Cell lines

HeLa, Human Embryonic Kidney 293T (293Ts), MDA-MB-231 and MCF7 were grown in Dulbecco’s modified Eagle’s medium (DMEM) supplemented with 10% foetal bovine serum (Gibco) and penicillin/streptomycin (Sigma Aldrich).

### Plasmid generation, siRNA Transfections and stable cell lines

USP11 cDNA sequence (USP11-201: ENST00000218348.7) was cloned into pHIV-zsGreen (a gift from Bryan Welm and Zena Werb; Addgene plasmid number 18121; RRID:Addgene_18121)^63^. siRNA sequences were synthesised by Sigma unless otherwise stated. siRNA transfections were carried out using Interferin (Polyplus) according to the manufacturer’s instructions, and all experiments were performed 72 h after the knockdown. Sequences are listed within Supplementary Table 1. Concentration utilised are: siCTRL (50 nM), siUSP11-pTRIPZ (50 nM), siUSP11-2 (50 nM); siPRMT1 (50 nM), siMRE11 (100 nM) and siCtIP (100 nM). Stable MCF7 cell lines expressing shCTRL (5′-CCTAAGGTTAAGTCGCCCTCG-3′) and shPRMT1 (5′-TGAGCGTTCCTAGGCGGTTTC-3′) have been described previously^64^.

siRNA-resistant USP11 constructs were generated by mutating the sequence from CCGTGATGATATCTTCGTCTA to C**A**G**G**GA**C**GA**C**AT**A**TT**T**GT**G**TA (for siUSP11-2) and from ACCTTAATCGGGTGAAGAA to ACCT**C**AA**CA**G**A**GT**T**AA**A**AA using the Q5 site-directed mutagenesis kit (NEB). MRE11 wildtype and MRE11 R/K constructs were obtained from Stephane Richard (McGill University, Canada) and subcloned into pHIV-dTomato (a gift from Bryan Welm; Addgene plasmid number 21374; RRID:Addgene_21374). To generate stable USP11-depleted cells, pTRIPZ-shUSP11 was obtained from Dharmacon (clone V2THS_41514) and used to generate lentiviral particles for cell infection followed by selection with 2 μg/ml puromycin. To enable expression of ectopic USP11-WT or R433K with concurrent depletion of endogenous USP11, cells were lentivirally infected with pHIV-zsGreen-HA-USP11-siRNA-resistant constructs. GFP positive populations were isolated by flow cytometry.

### Colony survival assays

Cells were plated at different densities according to the increasing doses of olaparib treatment 24 h before the addition of the drug. The DNA-damaging agent was replaced every three days and after 14 days cells were fixed and stained with 2% methylene blue in 50% ethanol. Colonies with more than 50 cells were considered as positive and the percentage of the surviving fraction was calculated based on the plating density and normalised to the untreated cells.

### G2/M checkpoint assay

Cells were treated with 5 Gy IR and harvested at the time points indicated. After fixation in 70% ethanol, cells were washed twice in PBS (0.1% Tween-20) and then incubated on a roller at 4 °C in ice-cold PBS (0.25% Triton-X 100) for 15 min. After washing with PBS (1% BSA), cells were incubated in 100 μl PBS (1% BSA) containing 1:1000 dilution of phospho-histone H3S10 antibody (CST: 9701) for 1 h followed by a 1 h incubation with anti-rabbit DyLIGHT 594 secondary antibody (Fisher: 10108403). Cells were washed once with PBS (1% BSA), then PBS, and then resuspended in FACS buffer (PBS minus Ca^2+^; 2 mM EDTA) supplemented with Vybrant DyeCycle Violet stain (Invitrogen: V35003). After 30 min incubation at 37 °C, cells were analysed by flow cytometry on a BD LSRFortessa X-20 using BD FACSDiva software (v.8.0.1). Representative FACS profiles were generated using FlowJo (v10.6.1) software.

### Immunoblotting

Cells were lysed in RIPA buffer (50 mM Tris pH7.4, 150 mM NaCl, 1 mM EDTA, 1% NP40, 0.5% sodium deoxycholate, 0.1% SDS, 10% glycerol, 1 mM PMSF, 50 mM NaF, 10 mM Na_3_VO_4_, 1 μg/ml leupeptin and 1μg/ml aprotinin) or in 0.1% NP40 lysis buffer (150 mM NaCl, 20 mM Tris pH 7.5, 0.5 mM EDTA, 1 mM Na_3_VO_4_, 50 mM NaF, 1 mM β-glycerophosphate, 100 mM PMSF, 10 μg/ml leupeptin, 10 μg/ml aprotinin). Cell extracts were sonicated twice followed by centrifugation to clarify lysate. Protein concentration was determined by Bradford Assay according to manufacturer’s instructions (BioRad). Protein lysates were resolved by sodium dodecyl-sulfate polyacrylamide gel electrophoresis (SDS-PAGE), transferred onto PVDF and incubated with primary antibody overnight, followed by HRP-linked secondary antibody for 1 h at room temperature. The signal was detected using ECL western blotting substrate (Pierce). Antibodies utilised include: USP11 (Bethyl: A301-613A; 1:2000); PRMT1 (CST: 24495; 1:1000); tubulin (Sigma: T6199; 1:5000), Flag-HRP (CST; 2044; 1:1000), P4D1 (Santa Cruz Biotechnology: sc-8017; 1:1000), H4R3me2a (Active Motif: 39705; 1:1000), actin (Sigma: A2228; 1:5000), p-Chk1-Ser-345 (CST: 2348; 1:1000), Chk1 (Santa Cruz: sc8408; 1:1000), p-Chk2-T68 (Abcam: Ab32148; 1:1000), Chk2 (CST: 6334; 1:1000), γH2AX (Millipore: 05-636; 1:1000), MRE11 (CST: 4895; 1:1000), CtIP (clone 14-1^65^; 1:1000), ADMA (Asym26) (Epicypher: 13281001; 1:1000), GFP (Sigma: 11814460001; 1:2000), HA (Biolegend: MMS-101R; 1:2000), cyclin A (Santa Cruz: sc-271682; 1:1000) and GAPDH (Abcam: ab9485; 1:5000).

### Immunoprecipitation

Myc-tagged PRMT1 and myc-MRE11 expressing cells were lysed in 0.1% NP40 lysis buffer (150 mM NaCl, 20 mM Tris pH 7.5, 0.5 mM EDTA, 100 mM PMSF, 10 μg/ml leupeptin, 10 μg/ml aprotinin), sonicated twice at 25% amplitude, and immunoprecipitated overnight with 2 μg anti-Myc (9E10) (Santa Cruz: sc-40). After extensive washing in NP-40 lysis buffer, complexes were eluted and analysed by SDS-PAGE and immunoblotting. Endogenous MRE11 was immunoprecipitated as in Vadnais et al.^18^. Briefly, cell lysates were trypsinised and lysed in buffer I, clarified by centrifugation and pellet resuspended in buffer. Totally, 2 μg of MRE11 antibody (ab109623) was added per sample and incubated at least for 3 h at 4 °C and protein complexes were captured with protein G sepharose beads. Complexes were washed four times with buffer II, before analysis by SDS-PAGE and immunoblotting.

### In vivo methylation assays

Transfected cells were cultured in methionine-free DMEM (Sigma Aldrich) supplemented with 10% foetal calf serum (FCS) and 1% glutamine. To inhibit de novo protein synthesis, cycloheximide (100 μg/ml) and chloramphenicol (40 μg/ml) were added for 1 h prior to labelling with l-[Methyl-^3^H]-methionine (specific activity 70–85 Ci (2.59–3.145 TBq)/mmol, 10 μCi/ml media; Perkin Elmer) for 4 h. When indicated, Ro-3306 (9 μM, Sigma Aldrich) was added 20 h post-tritium labelling, or cells exposed to 10 Gy X-ray irradiation. Cells were harvested and lysed in RIPA buffer (50 mM Tris pH 7.4, 150 mM NaCl, 1 mM EDTA, 1% NP40, 0.5% sodium deoxycholate 0.1% SDS, 10% glycerol, 1 mM PMSF, 50 mM NaF, 10 mM Na_3_VO_4_, 1 μg/ml leupeptin and 1 μg/ml aprotinin), and Flag or USP11 proteins immunoprecipitated overnight using anti-Flag M2-affinity beads (Sigma Aldrich) or anti-USP11 (Bethyl: A301-613A; 1 μg/ml), respectively. After five washes with RIPA buffer, immunoprecipitates were denatured, resolved by SDS-PAGE and transferred onto a nitrocellulose membrane. To verify equal immunoprecipitation, one-tenth of the immunoprecipitation was retained for Western blot analysis. To enhance tritium signal, membranes were treated with EN3HANCE (Perkin Elmer) and exposed to autoradiography film for 2–4 weeks at −80 °C.

### In vitro methylation assays

Immunoprecipitated or recombinant purified human USP11 were incubated with 1 μg recombinant GST-PRMT1 and 1 μl S-[methyl-^3^H]–adenosyl-l-methionine (specific activity 55–85 Ci/mmol, PerkinElmer) in PBS at 37 °C for 90 min and denatured protein resolved by SDS-PAGE. Protein was transferred onto nitrocellulose membrane, and tritium signal was enhanced by treating membranes with EN3HANCE (PerkinElmer). Membranes were exposed to autoradiography film for at least 1 month at −80 °C. Alternatively, after siUSP11 knockdown, cells were lysed in RIPA buffer (50 mM Tris pH 7.4, 150 mM NaCl, 1 mM EDTA, 1% NP40, 0.5% sodium deoxycholate 0.1% SDS, 10% glycerol, 1 mM PMSF, 50 mM NaF, 10 mM Na_3_VO_4_, 1 μg/ml leupeptin and 1 μg/ml aprotinin) and incubated with Flag-Agarose beads for 2 h at 4 °C. After four washes with RIPA buffer (including one wash with high salt (350 mM NaCl) RIPA) and one wash with PBS, Flag-tagged PRMT1 was eluted with 300 μg/ml of Flag peptide. For histone H4R3 methylation assay, 1 μg of H4 and 80 μM of SAM were mixed with the eluted Flag-PRMT1 and incubated at 37 °C for the indicated time.

### Deubiquitylating assay

In vitro deubiquitylating assay with Ub-AMC was performed by coupling the methylation and DUB reactions. First, 60 nM recombinant human His-USP11-WT, His-USP11-R433K or histone H4 was in vitro methylated by recombinant GST-PRMT1 (reaction containing 80 μM SAM, 150 mM NaCl and 150 mM Tris HCl pH 7.5). After 90 min methylation reaction, reactions were cooled to 22 °C, followed by digital dispensation (D300e TECAN) of 0.2–1 μM Ub-AMC to initiate the deubiquitylation reaction. Every experimental condition was repeated in technical triplicate and read in a kinetic loop for 90 min with a microplate reader (Spark TECAN). The initial velocities were plotted against the Ub-AMC concentrations and fitted to a Michaelis–Menten model using GraphPad Prism.

For ex vivo deubiquitylating assays, 293 T cells were transfected with siUSP11 and then transfected with the indicated Flag-tagged USP11 constructs, lysed in RIPA buffer (50 mM Tris pH 7.4, 150 mM NaCl, 1 mM EDTA, 1% NP40, 0.5% sodium deoxycholate, 0.1% SDS, 10% glycerol, 1 mM PMSF, 50 mM NaF, 10 mM Na_3_VO_4_, 1 μg/ml leupeptin and 1 μg/ml aprotinin) and then subjected to Flag immunoprecipitation/peptide elution. The eluted USP11 proteins were diluted in DUB assay buffer (50 mM Tris-HCl pH 7.5, 50 mM NaCl, 10 mM DTT) and incubated at 24 °C for 10 min before the reaction. For the DUB assays, 0.2 µg of di-ubiquitin (K63-linked) (Ubiquigent: 60-0107-010) was added for each reaction and incubated at 30 °C for the indicated time. Proteins were resolved by SDS-PAGE, transferred onto PVDF, incubated with denaturing buffer (6 M guanidine HCl, 1 mM DTT) for 30 min at 4 °C, washed extensively with TBS-Tween and then blocked for immunoblot analysis.

### Homology modelling of USP11

Homology modelling of the USP11 catalytic core domain was performed using the programme MODELLER^67^. This was based on USP11 sequence alignments with USP15 (PDB IDs 6GHA, 6CPM^40,41^, and USP4 (PDB ID 2Y6E^42^) crystals structures as templates (sequence identities of 62.72% with USP15 and 63.87% with USP4 to the target USP11, respectively).

### In vitro GST pull-down

In vitro GST pull-down was performed by incubating 1 μg of recombinant GST or GST-PRMT1 with glutathione 4B sepharose beads overnight at 4 °C in PBS/0.01% IGEPAL. Totally, 10% of the coupling reaction was taken as input and the rest was incubated with 2 μg of recombinant human USP11 for at least 3 h. Following 4 washes with PBS/0.01% NP40, samples were denatured, resolved by SDS-PAGE and immunoblotted.

### MultiDsk pull-down

Where indicated, cells were incubated with 9 μM Ro-3306 for 20 h prior to collection. Cells were lysed in 0.1% NP40 lysis buffer (150 mM NaCl, 20 mM Tris pH 7.5, 0.5 mM EDTA, 100 mM PMSF, 10 μg/ml leupeptin, 10 μg/ml aprotinin) supplemented with 100 mM N-ethylmaleimide (Sigma), sonicated 3 times and clarified lysate incubated with either wildtype or UBA mutated GST-MultiDsk^57^ for 3 h at 4 °C rotating. After 4 washes with lysis buffer, denatured samples were resolved by SDS-PAGE, transferred onto PVDF, incubated with denaturing buffer (6 M guanidine HCl, 1 mM DTT) for 30 mins at 4 °C, washed extensively with TBS-Tween and then blocked for immunoblot analysis.

### Flag-PRMT1 affinity purification for mass spectrometry

Flag-PRMT1 was cloned into pHIV-zsGreen and stable 293T cell lines were generated. Control 293T-pHIV-zsG or test 293T-pHIV-zsG-Flag-PRMT1 cells were lysed in 0.1% NP40 lysis buffer (150 mM NaCl, 20 mM Tris pH 7.5, 0.5 mM EDTA, 1 mM Na_3_VO_4_, 50 mM NaF, 1 mM β-glycerophosphate, 100 mM PMSF, 10 μg/ml leupeptin, 10 μg/ml aprotinin). After sonication, 3 mg of protein was precleared with protein G sepharose for 30 min at 4 °C and samples incubated with Flag-M2-agarose affinity beads (Sigma; A2220) for 2 h at 4 °C. Immunoprecipitations were washed three times with 0.1% NP40 lysis buffer and once with 10 mM Tris pH7.5/150 mM NaCl. Protein complexes were eluted twice with 50 μl Flag peptide (500 μg/ml; Sigma) using a thermoshaker set to 1200 pm, 15 °C for 30 min. Complexes were resolved by SDS-PAGE (NuPAGE Bis–Tris: ThermoFisher), fixed and stained with SYPRO Ruby as to the manufacture’s protocol (Lonza). Three independent biological experiments were conducted (i.e. Flag M2 immunoprecipitation from zsGreen or zsGreen-Flag-PRMT1 expressing 293 T cells), and denatured samples were stored before resolving by SDS-PAGE at the same time. Inclusion of Flag M2-affinity beads in pHIV-zsG expressing cells (empty vector) allowed identification of non-specific interactors. The threshold was set such that at least two spectral counts could be identified in at least one biological repeat of the test 293T-pHIV-zsG-Flag-PRMT1 cells. Proteins identified through low spectral counts (two or less) were only considered if the corresponding empty vector sample demonstrated zero spectral counts.

### Flag-USP11 and HA-USP11 affinity purification for methylation site identification by mass spectrometry

293T cells were transiently transfected with either HA or Flag-tagged USP11, and lysed in RIPA buffer (50 mM Tris pH7.4, 150 mM NaCl, 1 mM EDTA, 1% NP40, 0.5% sodium deoxycholate, 0.1% SDS, 10% glycerol, 1 mM PMSF, 50 mM NaF, 10 mM Na_3_VO_4_, 1 μg/ml leupeptin and 1 μg/ml aprotinin) or in 0.1% NP40 lysis buffer (150 mM NaCl, 20 mM Tris pH 7.5, 0.5 mM EDTA, 1 mM Na_3_VO_4_, 50 mM NaF, 1 mM β-glycerophosphate, 100 mM PMSF, 10 μg/ml leupeptin, 10 μg/ml aprotinin). Cell extracts were sonicated twice times followed by centrifugation to clarify lysate and incubated overnight with either anti-HA antibody (BioLegend MMS-101R) followed by capture with Protein G sepharose for 2 h, or Flag-M2-agarose affinity beads (Sigma; A2220). After ×4 washes with RIPA buffer, denatured proteins extracts were resolved by SDS-PAGE (NuPAGE Bis–Tris: ThermoFisher), left unfixed and stained with a MeOH-free ProtoBlue Safe Coomassie (S6-0044; Gene flow). Bands corresponding to the molecular weight of USP11 were excised and processed as below.

### Mass spectrometry

Each lane was divided into 10 Polyacrylamide gel slices, and proteins prepared for mass spectrometry analysis using the Janus liquid handling system (PerkinElmer, UK). Briefly, the excised protein gel pieces were placed in a well of a 96-well microtitre plate and destained with 50% v/v acetonitrile and 50 mM ammonium bicarbonate, reduced with 10 mM DTT and alkylated with 55 mM iodoacetamide. After alkylation, proteins were digested with 6 ng/μL endoproteinase Asp-N or trypsin (Promega, UK) overnight at 37 °C. The resulting peptides were extracted in 2% v/v formic acid, 2% v/v acetonitrile. The digest was analysed by nano-scale capillary LC–MS/MS using an Ultimate U3000 HPLC (ThermoScientific Dionex, San Jose, USA) to deliver a flow of approximately 300 nL/min. A C18 Acclaim PepMap100 5 µm, 100 µm × 20 mm nanoViper (ThermoScientific Dionex, San Jose, USA), trapped the peptides prior to separation on a nanoEase M/Z HSS C18 T3 100 Å, 1.8 μm, 75 μm × 250 mm column (Waters, UK). Peptides were eluted with a 60 min gradient of acetonitrile (2–35% acetonitrile 0–30 min, 35–45% acetonitrile 30–37 min, 45–80% acetonitrile 37–40 min, held at 80% acetonitrile for 5 min and then returned to 2% acetonitrile for column equilibration). The analytical column outlet was directly interfaced via a nano-flow electrospray ionisation source, with a hybrid quadrupole orbitrap mass spectrometer (Q-Exactive HF-X Orbitrap, ThermoScientific, San Jose, USA). Data-dependent analysis was carried out using a resolution of 60,000 for the full MS spectrum, with AGC 1e6, max IT 45 ms, mass range 350–1800 m/z in profile mode, followed by 12 MS/MS spectra (resolution 17,500). MS/MS scans were acquired using a normalised collision energy of 28 for higher-energy collisional dissociation (HCD), AGC 1e5, max IT 40 ms, data were acquired in centroid mode. Singly charged and unassigned charge states were excluded. LC–MS/MS data were then queried against a protein database (UniProt KB, Human Reviewed 2019) using the Mascot search engine programme v2.4.1 (Matrix Science, UK)^66^. Database search parameters were set with a precursor tolerance of 10 ppm and a fragment ion mass tolerance of 0.1 Da. One missed enzyme cleavage was allowed and variable modifications for oxidised methionine, carbamidomethyl cysteine, pyroglutamic acid, phosphorylated serine, threonine and tyrosine and methyl arginine were included. A minimum peptide length was set to five amino acids. MS/MS data were validated using the Scaffold programme (Proteome Software Inc., USA), with a minimum of two unique peptides and a peptide FDR of 2.4. All data were additionally interrogated manually. For methyl-peptide identification, thresholds were set to >95% probability (Scaffold software). Full Scaffold settings are provided in Supplementary Data 1.

### Immunofluorescence, microscopy and image analysis

Cells were plated on poly-d-lysine coated coverslips (50 μg/ml) (Sigma Aldrich) 24 h prior to treatment with ionising radiation. Coverslips were placed into ice-cold pre-extraction buffer for 7 min (10 mM PIPES pH 6.8, 300 mM sucrose, 20 mM NaCl, 3 mM MgCl_2_ 0.5% Triton-X 100), then fixed for 10 min in 3.6% PFA. After washing in phosphate-buffered saline (PBS) and 1 h block (10% FCS in PBS), cells were incubated in primary antibody overnight at 4 °C and secondary antibody at 1:1000 for 1 h at room temperature. Cells were washed three times with PBS and mounted onto glass slides with Prolong gold anti-fade reagent with DAPI (Life Technologies). Staining was assessed through eyepiece counting using a Zeiss Axiovision 3 immunofluorescent microscope and representative images were taken using ZEN Blue software (v2.3). A minimum of 50–150 cells was counted for each experimental repeat. Antibodies used include: γH2AX **(**Millipore: 05-636; 1:500), 53BP1 (Novus: NB100-304; 1:300), RAD51 (Millipore: PC130; 1:500), RPA (Millipore: NA18; 1:200), CENPF (CST: 58982; 1:1000) and mitosin (BD: 610768; 1:1000).

### Proximal ligation assay (PLA)

HeLa-pTRIPZ-shCTRL-myc MRE11 or HeLa-pTRIPZ-shUSP11-myc MRE11 were seeded on poly-d-lysine coated coverslips (50 μg/ml) (Sigma Aldrich), treated with doxycycline (2 μg/ml; 72 h) and S-phase cells labelled with EdU for 2 h (10 μM). Where indicated, cells were treated with 9 μM Ro-3306 for 20 h post collection. Cells were washed twice with ice-cold PBS and incubated with ice-cold pre-extraction buffer for 5 min on ice (10 mM PIPES pH 6.8, 300 mM sucrose, 20 mM NaCl, 3 mM MgCl_2_ 0.5% Triton-X 100), followed by fixation with 3.6% PFA (room temperature for 10 min). After three washes with ice-cold PBS, cells were blocked in 5% BSA/PBS at 4 °C overnight. Labelling of EdU positive cells was detected using Click-IT EdU Cell Proliferation Kit, AlexaFluro azide-488 (Thermo-Fisher; C10337) as to manufacturer’s instructions. Duolink PLA was conducted as to the manufacturer’s instructions (Merck-Sigma). Briefly, anti-myc (Abcam: ab-32) and Asym26 (Epicypher: 13281001), both diluted to 1:100 in 5% FCS/PBS, were incubated with cells for 1 h at room temperature. Plus and minus PLA probes were diluted 1:5 in 5% FCS/PBS and incubated at 37 °C for 1 h. Ligation (45 min at 37 °C) and amplification (110 min at 37 °C) were conducted using the Duolink In Situ Detection Reagents Red kit (Merck-Sigma: DUO92008). Coverslips were mounted using Prolong gold anti-fade reagent with DAPI (Life Technologies). Images were taken with a Nikon ECLIPSE E600 immunofluorescent microscope and Volocity software v4.1 (Improvision). At least 70 nuclei per condition in each independent experiment was scored using ImageJ (v1.50i).

For USP11-WT and USP11-R433K reconstitution experiments, stable HeLa-USP11-WT/dTom-myc-MRE11 or HeLa-USP11-R433K/dTom-myc-MRE11 (USP11 modified so resistant to siUSP11-2) were transfected with siUSP11-2 and 72hrs later incubated with EdU (10 μM) for 2 h before processing for PLA. For control PLA experiments, HeLa-dTom-myc-MRE11 cells were transfected with siPRMT1 and processing for PLA 72 h later.

### Quantitative PCR

RNA was extracted from cells using the Qiagen RNeasy mini kit, DNase treated (Ambion) and cDNA synthesised using Superscript III (Invitrogen) according to the manufacturer’s instructions. qPCR was performed using a Stratagene Mx3005P detection system with SYBR Green incorporation. Primers for *Gfi1* and *actin* are listed in Supplementary Table 1. All primer pairs spanned exon-intron boundaries and generated a single product as determined by dissociation curve analysis.

### Statistical analysis

Statistical analysis was carried out as indicated in the figure legend using either Student’s *t* test, 2 tailed, equal variance (Excel version 16.16.27), one-way ANOVA with Tukey post hoc test (GraphPad Prism 9) or Mann–Whitney (GraphPad Prism 9).

### Reporting summary

Further information on research design is available in the Nature Research Reporting Summary linked to this article.

## Supplementary information

Supplementary Information

Description of Additional Supplementary Files

Supplementary Data 1

Reporting Summary

## Data Availability

The raw and/or processed data underlying the bar charts, scatter graphs and uncropped gels generated in this study are provided in the Source Data file. The Flag-PRMT1 Mass Spectrometry data generated in this study are available have been deposited to the ProteomeXchange Consortium via the PRIDE partner repository with the dataset identifier PXD028324. Analysed Flag-PRMT1 Mass Spectrometry is presented in Supplementary Data 1. Data presented in Supplementary Fig. 1c was compiled from the CCLE (https://sites.broadinstitute.org/ccle/); data presented in Supplementary Fig. 7 was compiled from Kaplan–Meier Plotter (https://kmplot.com/analysis/) using the breast cancer mRNA gene chip database with probes 206445_s_at (PRMT1) and 208723_at (USP11). Structures used to model USP11 were derived from the Protein Data Bank in Europe (PDBe) using IDs 6GHA, 6CPM and 2Y6E. Other data that support the findings of this study are available from the corresponding authors upon reasonable request. Source data are provided with this paper.

## Source data

Source Data
