# Supplementary material for: Arginine methylation and ubiquitylation crosstalk controls DNA end-resection and homologous recombination repair
Source: Nat Commun. 2021 Nov 2;12:6313. doi: 10.1038/s41467-021-26413-6 (PMC8564520; doi:10.1038/s41467-021-26413-6)
Supplement: Supplementary file 1 — Supplementary Information [file 41467_2021_26413_MOESM1_ESM.pdf]

Supplementary Information

Arginine methylation and ubiquitylation crosstalk controls DNA end-resection and homologous recombination repair

Maria Pilar Sanchez-Bailon[1,4,6], Soo-Youn Choi[1,6], Elizabeth R. Dufficy[1,6], Karan Sharma[1], Gavin S. McNee[1], Emma Gunnell[2], Kelly Chiang[1], Debashish Sahay[1], Sarah Maslen[3], Grant S. Stewart[1], J. Mark Skehel[3,5], Ingrid Dreveny[2] and Clare C. Davies[1*]

[1] Institute of Cancer and Genomic Sciences, University of Birmingham, UK

[2] Biodiscovery Institute, School of Pharmacy, University of Nottingham, Nottingham, UK

[3] MRC Laboratory of Molecular Biology, Cambridge, UK

[4] Present address: Max Delbrück Center for Molecular Medicine, Berlin, Germany

[5] Present address: The Francis Crick Institute, London, UK.

[6]These authors contributed equally: Maria Pilar Sanchez-Bailon, Soo-Youn Choi, Elizabeth R. Dufficy.

* Corresponding author: c.c.davies@bham.ac.uk

Supplementary Figure 1

a

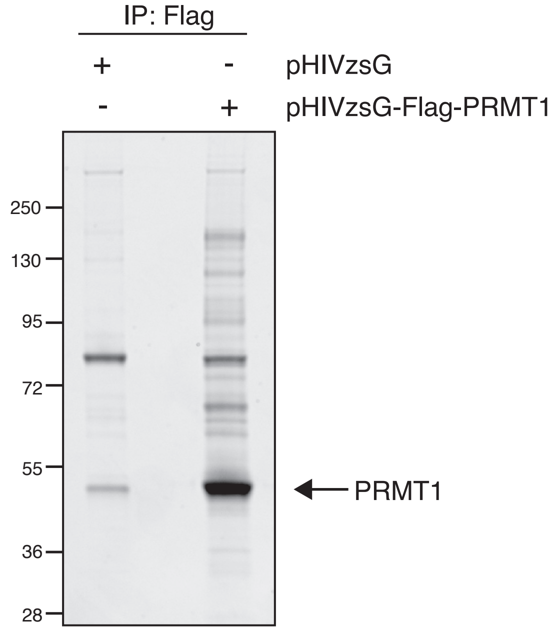

b

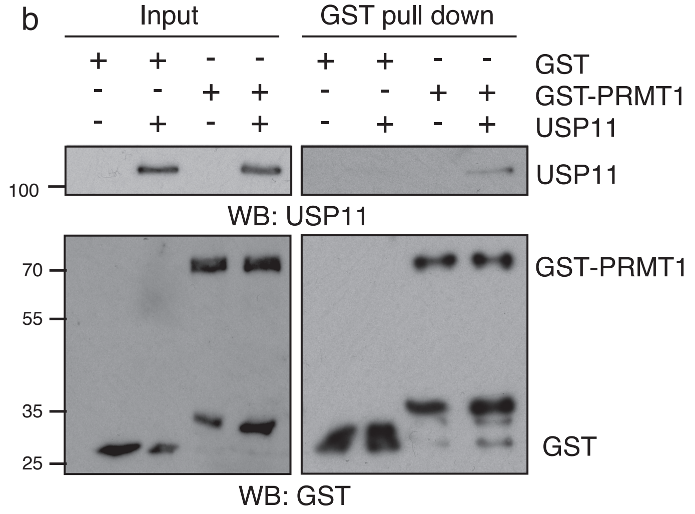

c

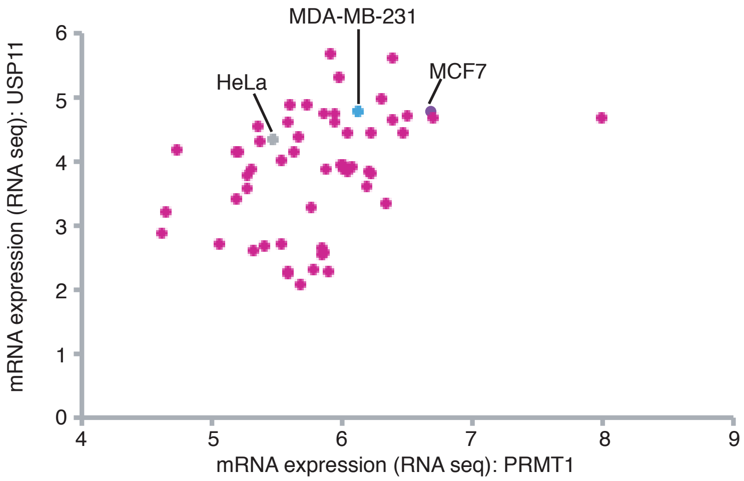

d

Experiment	No. methylated R433 peptides	Total no. peptides
A	1	10
B	2	19
C	2	12
D	1	5
E	1	5
F	1	3

e

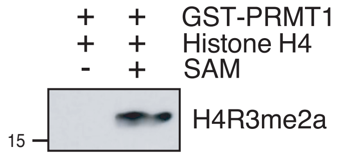

Supplementary Fig. 1: PRMT1 methylates USP11

(a) Mass spectrometry of Flag-PRMT1-associated proteins. 293T cells stably expressing empty vector (pHIV-zsG) or Flag-PRMT1 were subjected to M2-affinity purification and Flag-peptide elution before associated proteins were resolved by SDS-PAGE. Sypro ruby stain representative of three independent biological experiments. **(b)** USP11 and PRMT1 directly interact. GST-PRMT1 immobilised on Glutathione 4B Sepharose was incubated with recombinant USP11 and associated USP11 determined by immunoblotting. Representative image of 3 independent biological experiments. **(c)** mRNA expression of PRMT1 and USP11 in breast cancer and HeLa cells. Data taken from CCLE. **(d)** Table indicating the number of R433 methylated peptides identified within the total number of peptides spanning R433 in six independent experiments. **(e)** Validation of GST-PRMT1 methyltransferase activity using Histone H4 as substrate (associated with Fig. 3d). Representative image of 3 independent experiments. Uncropped blots provided within the Source Data file.

Supplementary Figure 2

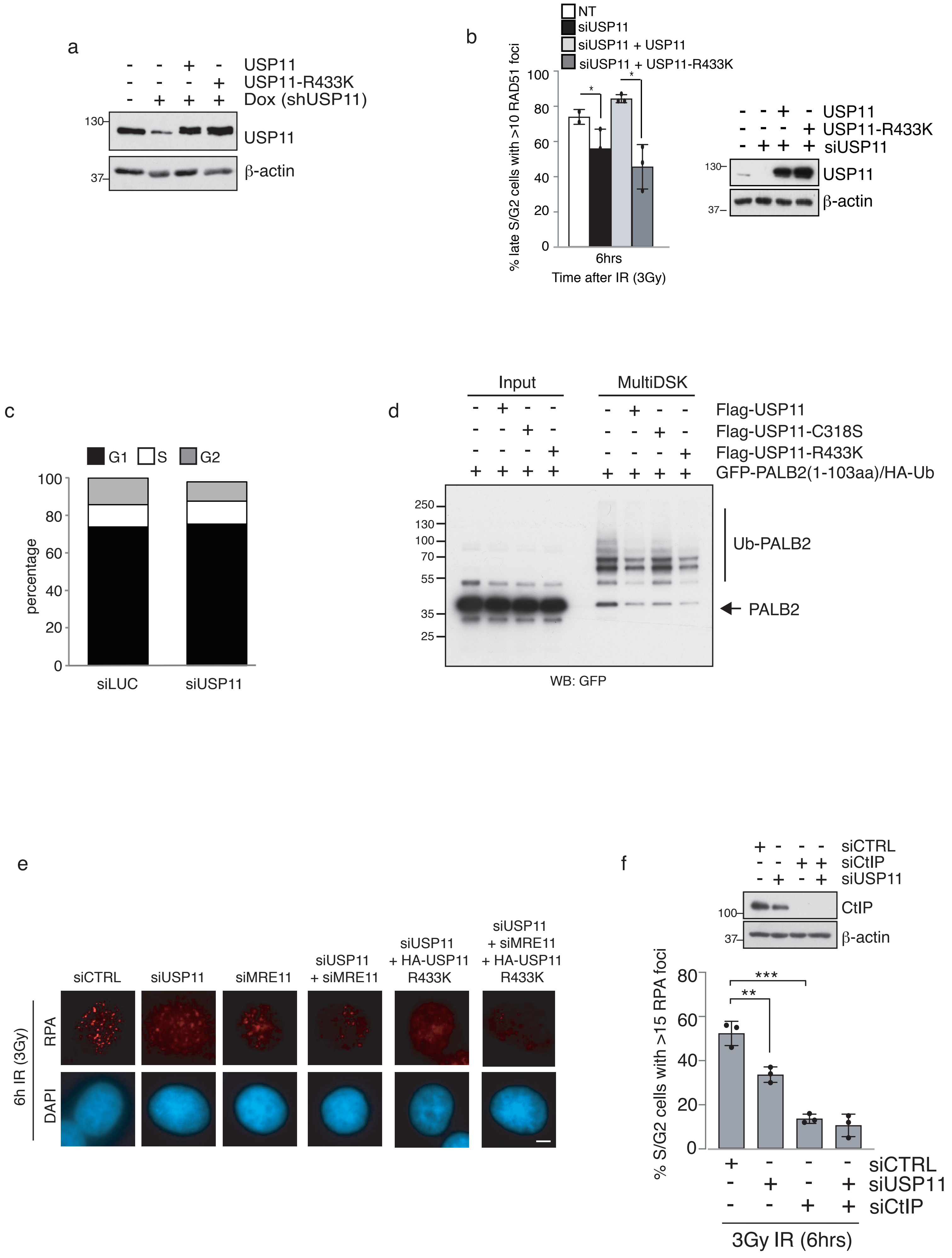

Supplementary Fig. 2: Methylation of USP11 at R433 regulates DNA repair

(a) Generation of MCF7 cells stably expressing doxycycline-inducible knockdown of USP11 in conjunction with ectopic expression of USP11 or USP11-R433K (1μg/ml doxycycline for 72hrs). Representative image of >3 independent experiments. **(b)** Knockdown and reconstitution of HeLa cells with USP11-R433K suppresses RAD51 foci formation in S/G2 cells after IR-induced DNA damage. HeLa cells stably expressing USP11 or USP11-R433K were transfected with siUSP11, exposed to 3Gy IR, and harvested 6 hrs later. Mitosin-positive cells were scored for RAD51 foci formation (mean ± SD; n=3 independent experiments; Student's *t*-test (two-sided, equal variance) *p=0.05 (NT versus siUSP11); *p=0.01 (USP11 versus USP11-R433K)). **(c)** Cell cycle analysis of HeLa cells transfected with siUSP11. **(d)** Methylation of USP11 is not required for the deubiquitylation of PALB2. 293T cells were transfected with the indicated constructs and ubiquitylated GFP-PABL2 (1-103aa) was isolated through MulitDSK affinity purification. Representative image of 2 independent experiments. **(e)** Representative immunofluorescence images to accompany Figure 5C. Scale bar represents 5μm. **(f)** USP11 is not epistatic with CtIP in the regulation of RPA foci formation after IR (3Gy) in late S/G2 MCF7 cells (mean ± SD; n=3 independent experiments; Student's *t*-test (two-sided, equal variance) **p=0.01; ***p=0.0003). Uncropped blots, raw and processed graphical data provided as a Source Data file.

Supplementary Figure 3

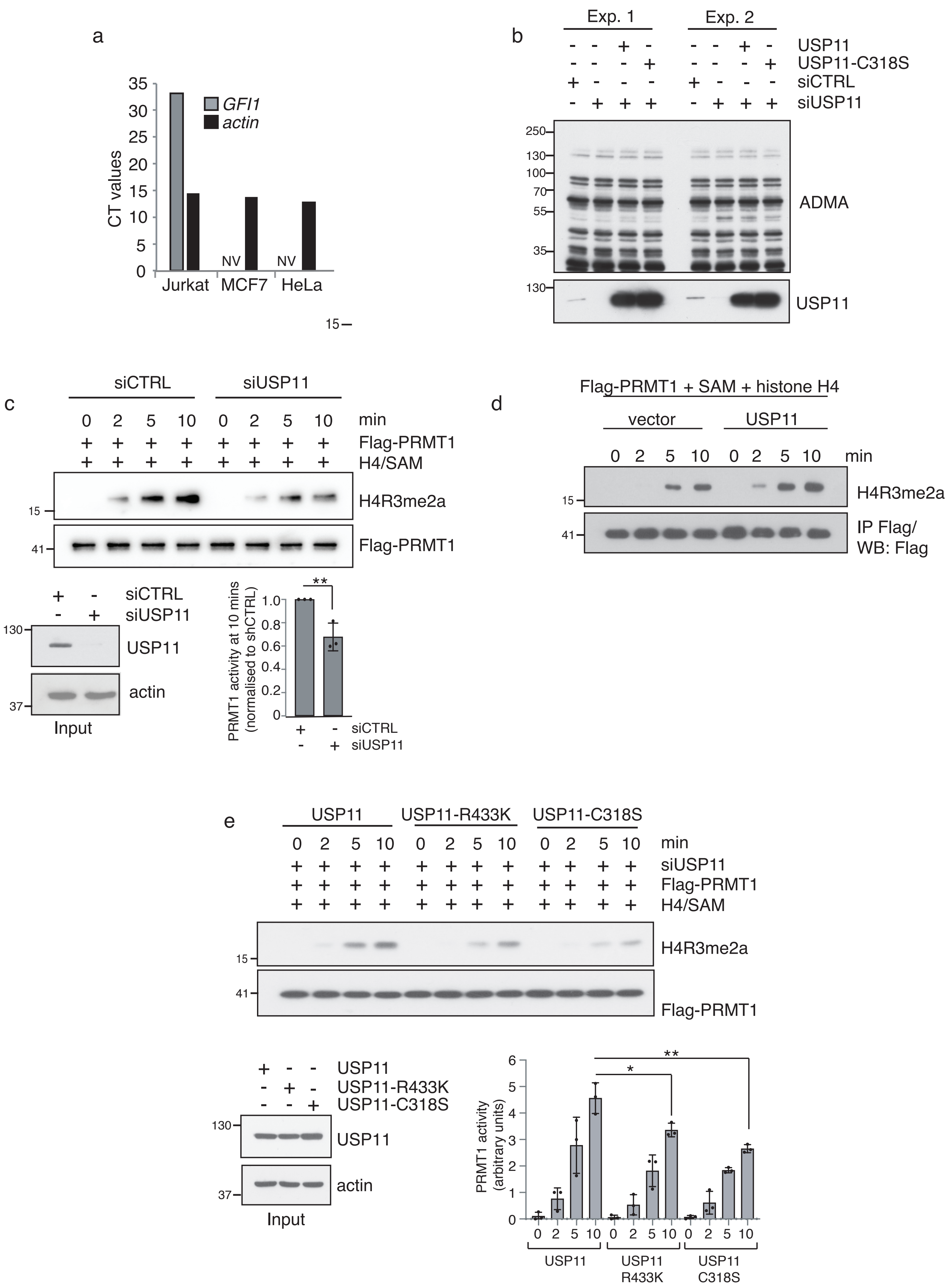

Supplementary Fig. 3: Interplay between USP11 and arginine methylation

(a) *GFI1* is not expressed in MCF7 or HeLa epithelial cells. qPCR data expressed as CT values. NV = No Value. **(b)** Knockdown of USP11 in stable Hela-USP11 or USP11-C318S cells does not change global ADMA levels. Image of two independent experiments. **(c)** Depletion of USP11 reduces PRMT1 activity. HeLa cells were transfected with siUSP11 and Flag-PRMT1 and 72 hrs later, PRMT1 immunoprecipitated for *in vitro* methylation assay using histone H4 as substrate. PRMT1 activity was determined through immunoblotting of histone H4R3 asymmetric dimethylation (H4R3me2a). Graph represents data at the 10 mins time point (mean ± SD; n=3 independent experiments; Student's *t*-test (two-sided, equal variance) **p =0.009). **(d)** Overexpression of USP11 increases PRMT1 activity. HeLa cells were transfected with pIRES2-USP11 and Flag-PRMT1 and 48 hrs later PRMT1 activity determined. Image of two independent experiments. **(e)** Expression of methyl-deficient or catalytically inactive USP11 reduces PRMT1 activity. HeLa cells were transfected with siUSP11, Flag-PRMT1 and USP11 constructs as indicated, 72 hrs later PRMT1 immunoprecipitated for *in vitro* methylation assay, and PRMT1 activity determined. Graph represents data of three independent experiments (mean ± SD; n=3 independent experiments; Student's *t*-test (two-sided, equal variance) *p =0.03; **p=0.005). Uncropped blots, raw and processed graphical data provided as a Source Data file.

Supplementary Figure 4

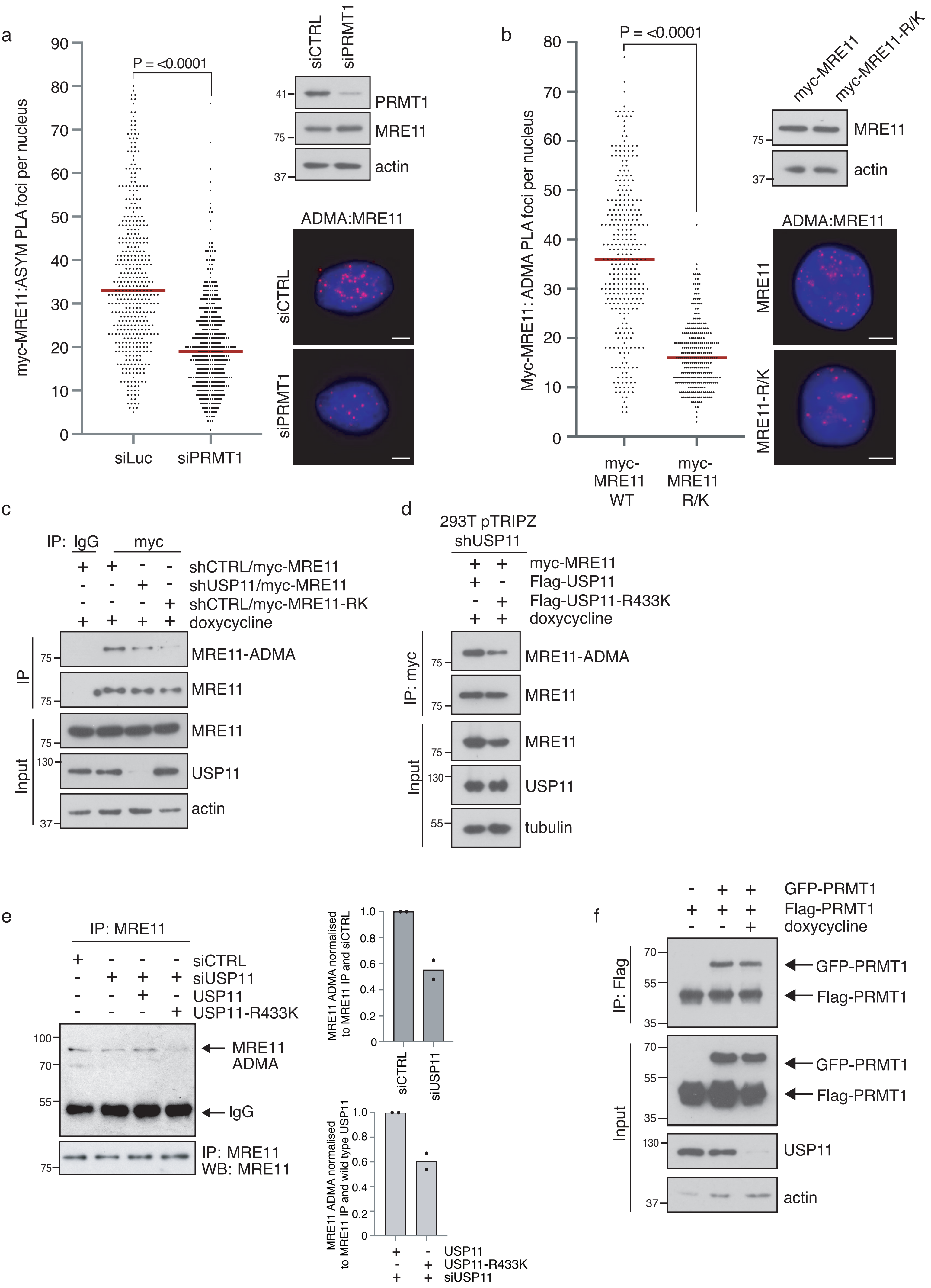

Supplementary Fig. 4: USP11 regulates MRE11 methylation

(a) Depletion of PRMT1 reduces MRE11 methylation as determined by PLA (antibodies directed towards myc-tagged MRE11 and ADMA). Data is from two independent experiments, with >150 nuclei scored per independent experiment (median PLA foci for siCTRL and siPRMT1 were 33 and 19 respectively; Mann-Whitney n_1= 430; n_2=414; p<0.0001; two-tailed). Scale bar represents 5μm. **(b)** Expression of myc-MRE11 mutated at arginine residues methylated by PRMT1 (MRE11-R/K) reduces MRE11 methylation as determined by PLA. Data is from two independent experiments, with >150 nuclei scored per independent experiment (median PLA foci for MRE11 wt and MRE11-R/K were 36 and 16 respectively; Mann-Whitney n_1= 321; n_2=301; p<0.0001; two-tailed). Scale bar represents 5μm. **(c)** Knockdown of USP11 reduces MRE11 methylation. HeLa-myc-MRE11 wildtype or MRE11 methyl-deficient (MRE11-R/K) cells stably expressing doxycycline inducible shUSP11 were treated with doxycycline for 48 hrs and MRE11 immunoprecipitated with a myc antibody. Levels of MRE11 methylation (MRE11-ADMA) were determined using Asym26 antibody. Representative image of 3 independent experiments. **(d)** Expression of USP11-R433K reduces ectopic MRE11 methylation. 293T cells stably expressing doxycycline inducible shUSP11 were transiently transfected with myc-MRE11, Flag-USP11 or Flag-USP11 R433K. After 48 hrs, cells were collected and MRE11 methylation determined by immunoprecipitation/immunoblotting. Representative image of 3 independent experiments. **(e)** Expression of USP11-R433K or USP11 knockdown decreases endogenous MRE11 methylation. MCF7-USP11 or MCF7-USP11-R433K cells were transfected with siUSP11 and endogenous MRE11 immunoprecipitated. Associated arginine methylation of MRE11 was detected by immunoblotting with a pan-ADMA antibody. Quantification is of 2 independent experiments. Dark grey bars represent MRE11 ADMA levels after USP11 knockdown; light grey bars represent MRE11 ADMA levels after re-expression of wildtype or methyl-deficient USP11. **(f)** Depletion of USP11 does not affect PRMT1 dimerisation. 293T cells stably expressing doxycycline inducible shUSP11 were transiently transfected with GFP-PRMT1 and Flag-PRMT1. After 48 hrs, cells were collected and Flag-PRMT1 immunoprecipitated. Associated Flag and GFP-tagged PRMT1 was detected by anti-PRMT1 antibodies. Representative image of 2 independent experiments. Uncropped blots, raw and processed graphical data provided as a Source Data file.

Supplementary Figure 5

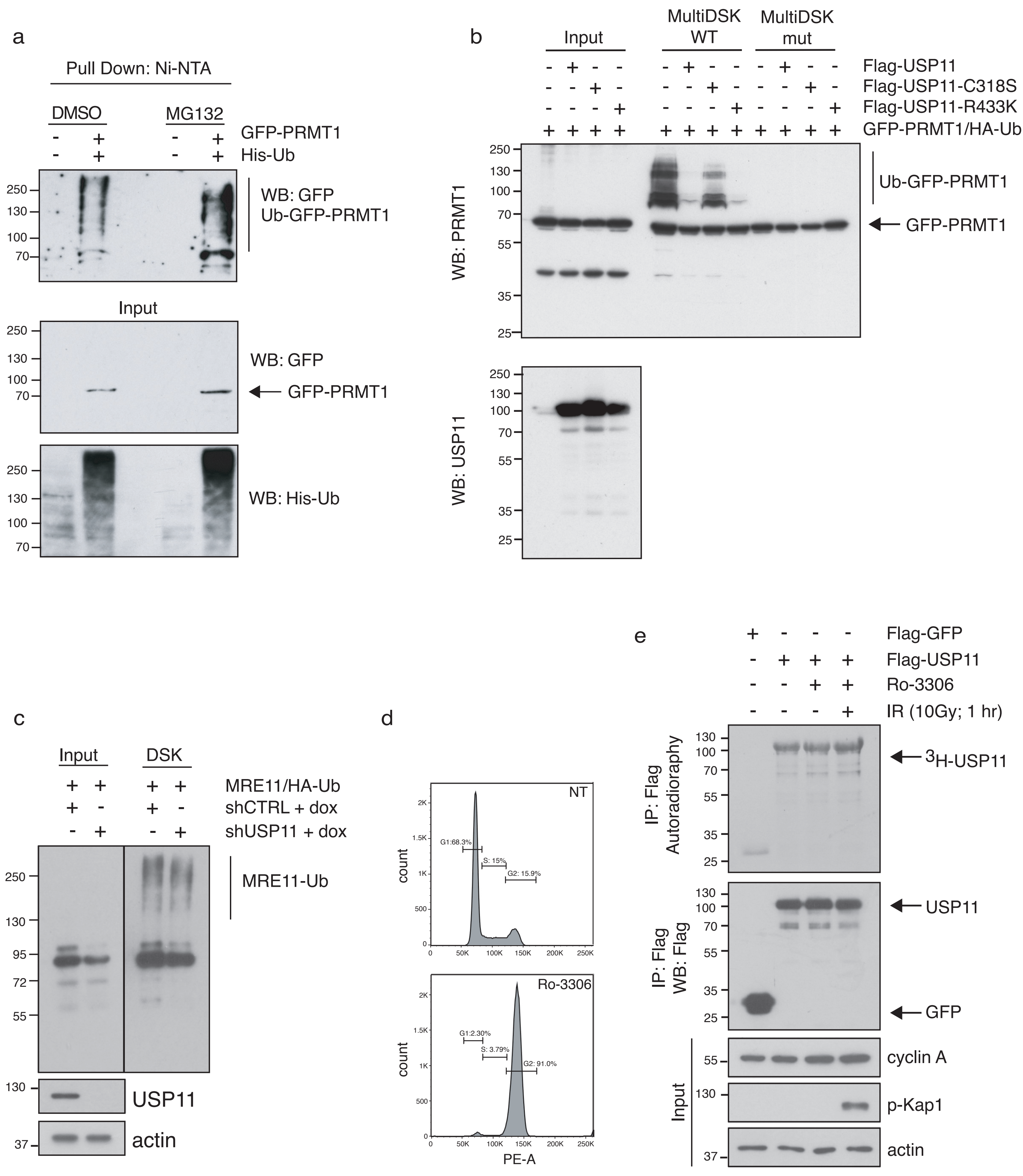

Supplementary Fig. 5: PRMT1 is a ubiquitylated protein

(a) PRMT1 is a ubiquitylated protein as determined by affinity purification of His-tagged ubiquitin in denaturing lysis conditions, and immunoblotting for associated proteins. Representative image of 2 independent experiments. **(b)** Ubiquitylation of PRMT1 in asynchronous 293T cells after expression of wild-type, catalytically inactive (C318S) and methyl-deficient (R433K) USP11 constructs. Ubiquitylated proteins were captured by recombinant MultiDSK protein and associated PRMT1 detected by immunoblotting. Specificity of ubiquitin binding is controlled through pulldown analysis using mutated MultiDSK that is unable to bind ubiquitin. Representative image of 3 independent experiments. **(c)** USP11 does not deubiquitylate MRE11, as determined by MultiDSK pulldown of MRE11 transfected into 293T-pTRIPZ-shCTRL or shUSP11 cells. Cells were treated with 2μg/ml doxycycline for 48 hrs prior to collection. Representative image of 2 independent experiments. **(d)** Propidium iodide staining and FACS analysis of HeLa cells treated with Ro-3306 (9μM; 20 hrs) demonstrating G2 cell cycle arrest. NT = no treatment. **(e)** Methylation of Flag-USP11 transiently transfected into 293T cells does not change in G2 arrested cells, or after IR-induced DNA damage. Methylation determined by ^{3}[H]-methionine labelling of cells and *in vivo* methylation assay. Uncropped blots provided as a Source Data file.

Supplementary Figure 6

a

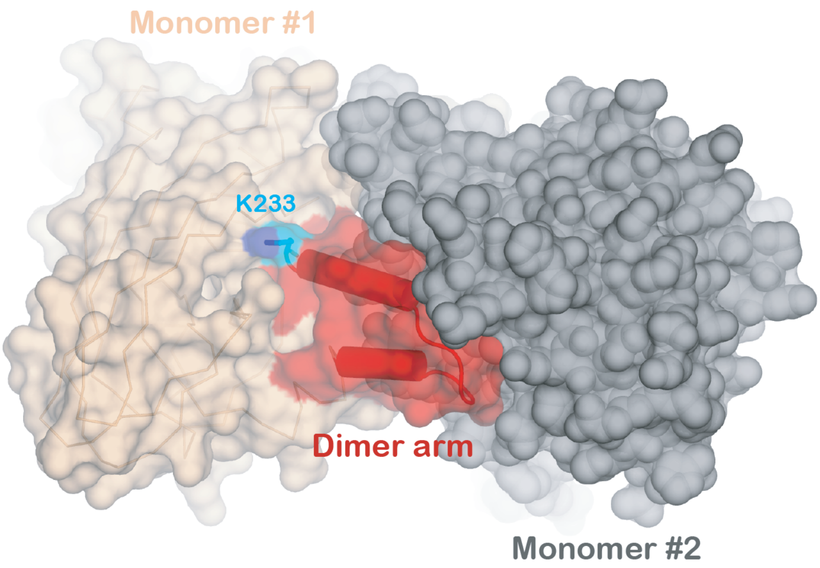

b

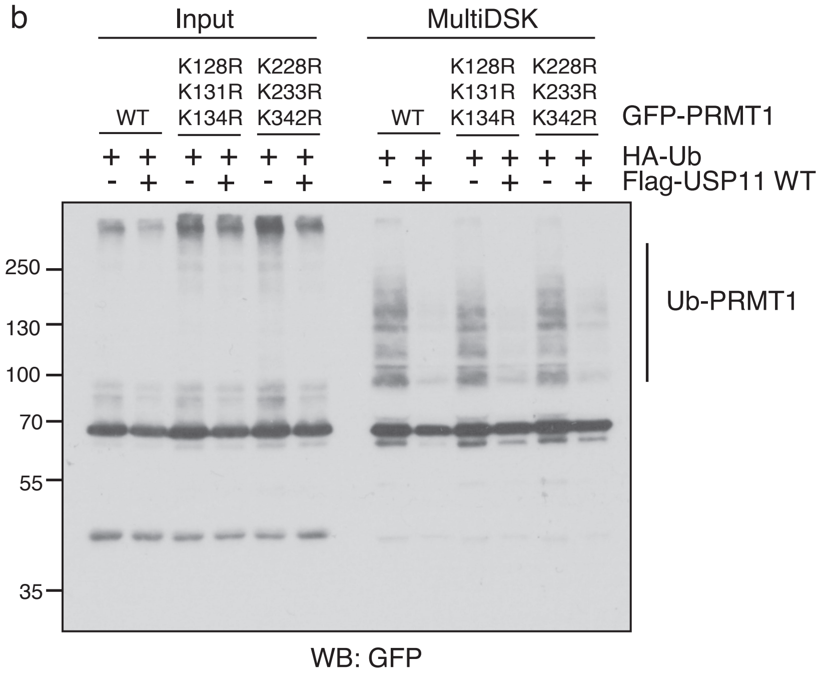

c

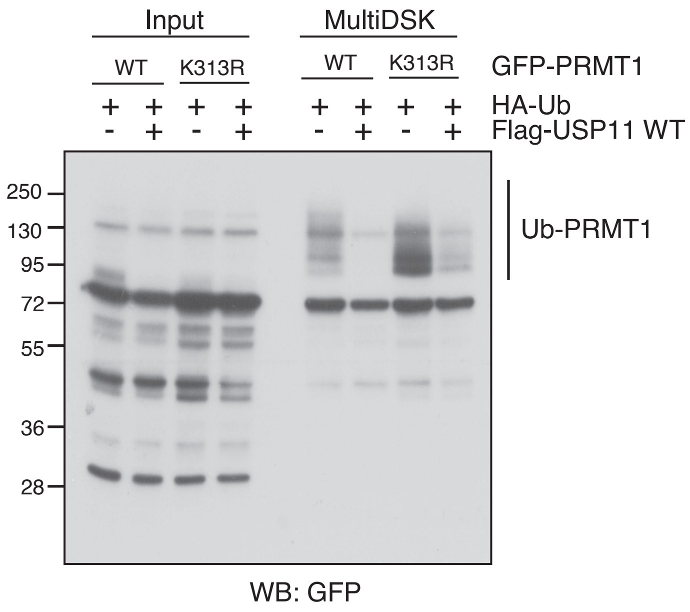

Supplementary Fig. 6: Attempts to map ubiquitylation sites on PRMT1 targeted by USP11

(a) Position of K233 (identified 18 times by Phosphosite Plus) located at the base of the dimer arm (modelled using structure 6NT2). (**b** and **c)** Ubiquitylation of GFP-PRMT1 constructs mutated in putative ubiquitin sites, and the effects of USP11 overexpression. Ubiquitylation of PRMT1 was detected by MultiDSK pulldown. Representative image of 2 independent experiments.

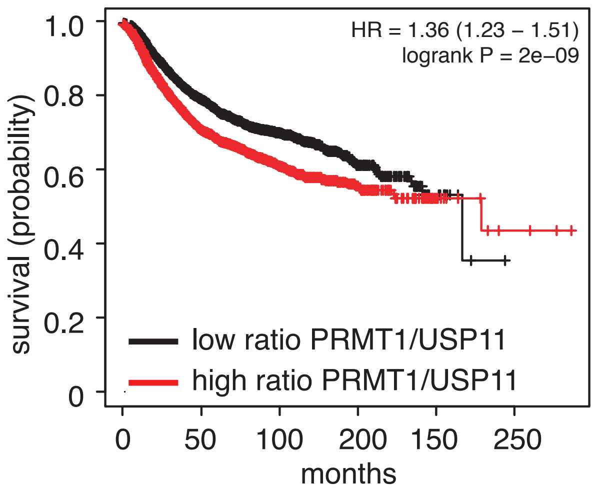

Supplementary Fig. 7: Breast cancer patients with high ratio of *PRMT1:USP11* transcripts have reduced survival rates

Data generated using kmplot.com on a dataset of 4929 patients using PRMT1 (206445_s_at) and USP11 (208723_at) probes respectively. Logrank *P* = 2e^{-9}; (HR (95% CI) = 1.36 (1.23-1.51), as determined by Kaplan-Meier survival estimate.

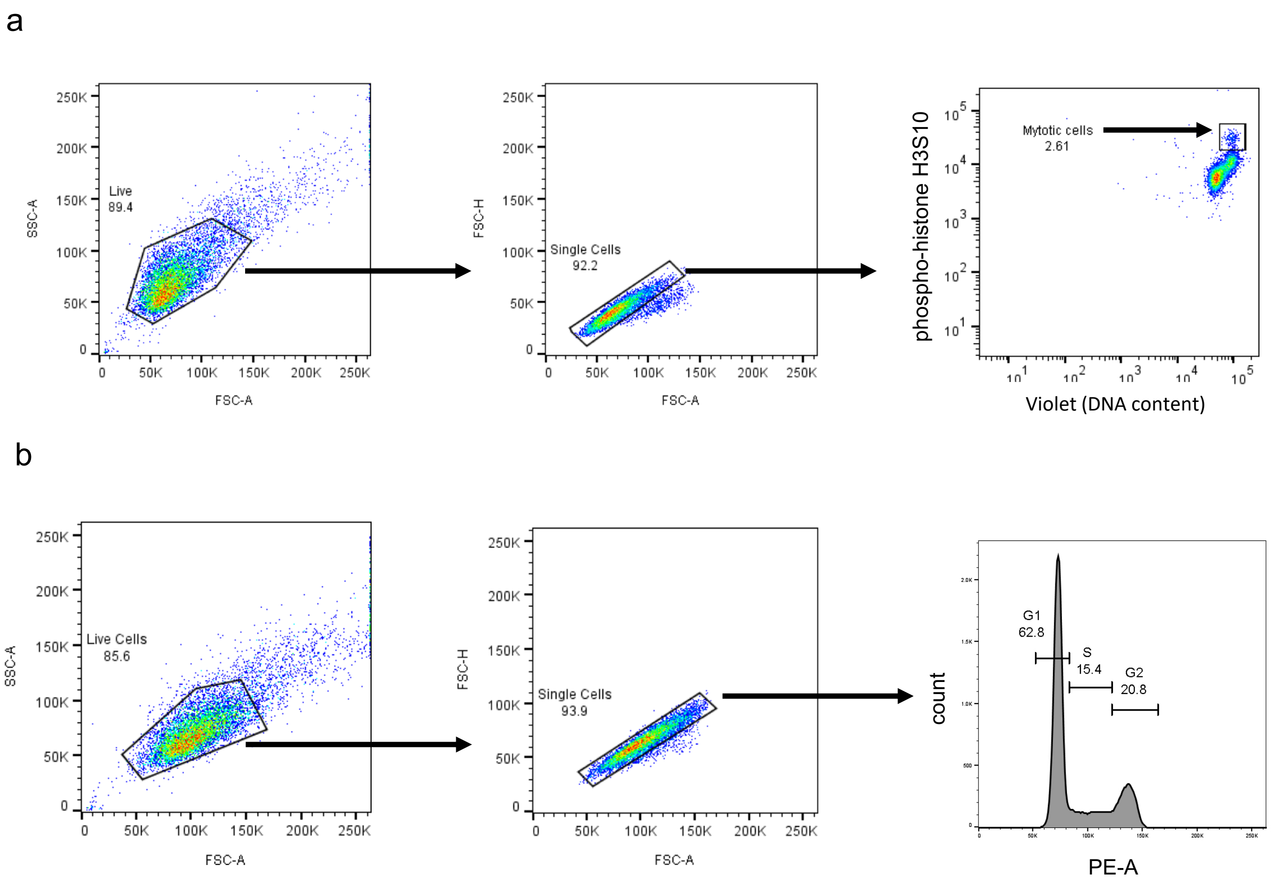

Supplementary Fig. 8: Gating strategies for flow cytometry analysis

(a) Analysis of proportion of mitotic cells, as measured by detection of Histone H3 Ser10 phosphorylation (data related to Fig. 5f). **(b)** Analysis of proportion of G1/S/G2 cells, as measured by DNA content (data related to Supplementary Fig. 5d).

Supplementary Table 1: Primers and siRNA sequences

siRNA and shRNA sequences	
siCTRL	5'-CGUACGCGGAAUACUUCGA-3'
siUSP11-pTRIPZ	5'-ACCUUAAUCGGGUGAAGAA-3'
siUSP11-2	5'-CCGUGAUGAUAUUUCGUCUA-3'
siCtIP	5'-GCUAAAACAGGAACGAAUC-3'
siPRMT1	5'-UGAGCGUUCCUAGGCGGUUUC-3'
siMRE11	SMARTpool, Horizon/Dharmacon (L-009271-00-0005)
shCTRL	5'-CCTAAGGTTAAGTCGCCCTCG-3'
shPRMT1	5'-TGAGCGTTCCTAGGCGGTTTC-3

quantative PCR primers (SYBR Green method)	
actin (human)	Forward: 5'-CTCTTCCAGCCTTCCTTCCT-3'<br>Reverse: 5'-GAAGTGTGACGTGGACATCC -3'
Gfi1 (human)	Forward: 5'-GCCCACCTCTTCCAAATTTAACC-3'<br>Reverse: 5'-CCGGAGGAGACCTTGACAC-3'
