# Supplementary material for: Arginine methylation and ubiquitylation crosstalk controls DNA end-resection and homologous recombination repair
Source: Nat Commun. 2021 Nov 2;12:6313. doi: 10.1038/s41467-021-26413-6 (PMC8564520; doi:10.1038/s41467-021-26413-6)
Supplement: Supplementary file 2 — Description of Additional Supplementary Files [file 41467_2021_26413_MOESM2_ESM.docx]

# **Arginine methylation and ubiquitylation crosstalk controls DNA end-resection and homologous recombination repair**

Maria Pilar Sanchez-Bailon, Soo-Youn Choi, Elizabeth R. Dufficy, Karan Sharma, Gavin S. McNee, Emma Gunnell, Kelly Chiang, Debashish Sahay, Sarah Maslen, Grant S. Stewart, J. Mark Skehel, Ingrid Dreveny and Clare C. Davies

**Supplementary Data 1.** Proteomics dataset from 293T cells stably expressing empty vector or Flag-PRMT1 after Flag-M2-affinity purification and Flag-peptide elution. Data represents total spectral counts from 3 independent biological experiments.
