# Supplementary material for: Arginine methylation and ubiquitylation crosstalk controls DNA end-resection and homologous recombination repair
Source: Nat Commun. 2021 Nov 2;12:6313. doi: 10.1038/s41467-021-26413-6 (PMC8564520; doi:10.1038/s41467-021-26413-6)
Supplement: Supplementary file 4 — Reporting Summary [file 41467_2021_26413_MOESM4_ESM.pdf]

nature portfolio

Corresponding author(s): Clare Davies

Last updated by author(s): Aug 26, 2021

Reporting Summary

Nature Portfolio wishes to improve the reproducibility of the work that we publish. This form provides structure for consistency and transparency in reporting. For further information on Nature Portfolio policies, see our Editorial Policies and the Editorial Policy Checklist.

Statistics

For all statistical analyses, confirm that the following items are present in the figure legend, table legend, main text, or Methods section.

n/a	Confirmed	
☐	☒	The exact sample size (*n*) for each experimental group/condition, given as a discrete number and unit of measurement
☐	☒	A statement on whether measurements were taken from distinct samples or whether the same sample was measured repeatedly
☐	☒	The statistical test(s) used AND whether they are one- or two-sided<br>*Only common tests should be described solely by name; describe more complex techniques in the Methods section.*
☒	☐	A description of all covariates tested
☒	☐	A description of any assumptions or corrections, such as tests of normality and adjustment for multiple comparisons
☐	☒	A full description of the statistical parameters including central tendency (e.g. means) or other basic estimates (e.g. regression coefficient) AND variation (e.g. standard deviation) or associated estimates of uncertainty (e.g. confidence intervals)
☐	☒	For null hypothesis testing, the test statistic (e.g. *F*, *t*, *r*) with confidence intervals, effect sizes, degrees of freedom and *P* value noted<br>*Give P values as exact values whenever suitable.*
☒	☐	For Bayesian analysis, information on the choice of priors and Markov chain Monte Carlo settings
☒	☐	For hierarchical and complex designs, identification of the appropriate level for tests and full reporting of outcomes
☒	☐	Estimates of effect sizes (e.g. Cohen's *d*, Pearson's *r*), indicating how they were calculated

Our web collection on statistics for biologists contains articles on many of the points above.

Software and code

Policy information about availability of computer code

Data collection	Q-Exactive HF-X Orbitrap, Thermoscientific<br>BD LSR Fortessa X-20<br>BD FACS Aria<br>Microplate reader (Spark TECAN)<br>Nikon ECLIPSE E600 immunoflourescence microscope<br>Zeiss Axiovert 3 immunoflourescence microscope
Data analysis	Mascot version 2.4.1 (Matrix Science, UK)<br>Scaffold4 version 4.8.1 (Proteome Software Inc., USA)<br>Microsoft Excel version 16.16.27<br>GraphPad Prim9 version 9.2.0<br>BD FacsDIVA v8.01 (Becton Dickinson)<br>FlowJo version 10.6.1<br>Volocity Software v4.1 (Improvision)<br>Image J (1.50i)<br>ZEN (Blue v2.3)<br>MODELLER version 10.1

For manuscripts utilizing custom algorithms or software that are central to the research but not yet described in published literature, software must be made available to editors and reviewers. We strongly encourage code deposition in a community repository (e.g. GitHub). See the Nature Portfolio guidelines for submitting code & software for further information.

Data

Policy information about availability of data

All manuscripts must include a data availability statement. This statement should provide the following information, where applicable:

- Accession codes, unique identifiers, or web links for publicly available datasets
- A description of any restrictions on data availability
- For clinical datasets or third party data, please ensure that the statement adheres to our policy

The raw and/or processed data underlying the bar charts, scatter graphs and uncropped gels generated in this study are provided in Source Data file. The Flag-PRMT1 Mass Spectrometry data generated in this study are available in the ProteomeXchange Consortium via the PRIDE partner repository with the dataset identifier PXD028324 [http://www.ebi.ac.uk/pride/archive/projects/PXD028324]. Analysed Flag-PRMT1 Mass Spectrometry is presented in Supplementary Table 1. Data presented in Supplementary Fig. 1c was compiled from the CCLE (https://sites.broadinstitute.org/ccle/); data presented in Supplementary Fig. 7 was compiled from Kaplan-Meier Plotter (https://kmplot.com/analysis/) using the breast cancer mRNA gene chip database with probes 206445_s_at (PRMT1) and 208723_at (USP11). Structures used to model USP11 were derived from the Protein Data Bank in Europe (PDBe) using IDs 6GHA, 6CPM and 2Y6E. Other data that support the findings of this study are available from the corresponding authors upon reasonable request.

Field-specific reporting

Please select the one below that is the best fit for your research. If you are not sure, read the appropriate sections before making your selection.

- [x] Life sciences
- [ ] Behavioural & social sciences
- [ ] Ecological, evolutionary & environmental sciences

For a reference copy of the document with all sections, see nature.com/documents/nr-reporting-summary-flat.pdf

Life sciences study design

All studies must disclose on these points even when the disclosure is negative.

Sample size	No statical tests were performed to determine sample size. Sample size for each experiment is indicated int he legend. Sample size was based on previous experimental experience using similar studies and assays employed within the field (Clarke et al., Mol. Cell, 2017 PMID: 28238654) (Wijnhoven et al., Mol. Cell, 2015 PMID: 26455393).
Data exclusions	No data was excluded from this study.
Replication	Number of independent biological experiments are stated in the legends. Experiments were conducted as at least three independent repeats, with the exception of Supplementary Fig. 4a-b (n=2), Supplementary Figure 5d (n=1 to test functionality of Ro-3306), and Supplementary Fig. 5e (n=2). All attempts of replications of experimental conditions were successful.
Randomization	All samples used in this study, including cultured cells, were allocated randomly to each condition.
Blinding	Immunoflourescence foci were counted in a blinded manner. Samples for immunoblotting and biochemical assays, or the addition of compounds, were known to the experimenter when preparing samples or setting up assays.

Reporting for specific materials, systems and methods

We require information from authors about some types of materials, experimental systems and methods used in many studies. Here, indicate whether each material, system or method listed is relevant to your study. If you are not sure if a list item applies to your research, read the appropriate section before selecting a response.

Materials & experimental systems

n/a	Involved in the study	
[ ]	[x]	Antibodies
[ ]	[x]	Eukaryotic cell lines
[x]	[ ]	Palaeontology and archaeology
[x]	[ ]	Animals and other organisms
[x]	[ ]	Human research participants
[x]	[ ]	Clinical data
[x]	[ ]	Dual use research of concern

Methods

n/a	Involved in the study	
[x]	[ ]	ChIP-seq
[ ]	[x]	Flow cytometry
[x]	[ ]	MRI-based neuroimaging

Antibodies

Antibodies used	Flag-M2 agarose affinity beads (Sigma: A2220)<br>Phospho-Histone H3 Ser 10 (Cell Signaling Technology: 9701)

USP11 (Bethyl: A301-613A)
PRMT1 (Cell Signaling Technology: 24495)
Tubulin (Sigma: T6199)
Flag-HFP (Cell Signaling Technology: 2044)
P4D1 (Santa Cruz: sc-8017)
H4R3me2a (Active Motif: 39705)
Actin (Sigma A2228)
Phospho-Chk1 Ser354 (Cell Signaling Technology: 2348)
Chk1 (Santa Cruz: sc-8408)
Phospho-Chk2 Thr68 (Abcam: Ab32148)
Chk2 (Cell Signaling Technology: 6334)
Gamma H2AX (Millipore: 05-636)
MRE11 (Cell Signaling Technology: 4895)
MRE11 (Abcam: ab109623)
CtIP (clone 14-1 originally from the lab of Richard Baer but provided by Prof. Grant Stewart)
ADMA-Asym26 (Epicypher: 13281001)
53BP1 (Novas: NB100-304)
Rad51 (Millipore: PC130)
RPA (Millipore: NA18)
CENPF clone D6X4L (58982)
Mitosin clone 11 (BD Biosciences: 610768)
c-myc 9E10 (Abcam: Ab32)
c-myc 9E10 (Santa-Cruz: sc-40)
GFP clone 7.1/13.1 (Sigma: 11814460001)
HA clone 16B12 (Biolegend: MMS-101R)
cyclin A (Santa Cruz: sc-271682)
GAPDH (Abcam: ab9485)

Validation

All the antibodies, with the exception of CtIP (Yu, X. & Baer, R. Nuclear Localization and Cell Cycle-specific Expression of CtIP, a Protein That Associates with the BRCA1 Tumor Suppressor. J Biol Chem 275, 18541–18549 (2000)), are from commercial sources and have been validated by the vendors (see links below).

In this study, we have validated the following antibodies:
USP11, PRMT1, MRE11, CtIP were confirmed through siRNA knockdown. ADMA-Asym26 was confirmed through knockdown of PRMT1 and expression of methyl-deficient MRE11. gamma H2AX, 53BP1, RPA, Rad51, Phospho-Chk1, Phospho-Chk2 antibodies were validate by their known formation of foci or induction after DNA damage induced by ionising radiation. Flag antibodies and affinity resin was confirmed through inclusion of empty vector samples. Cyclin A was validated by its induction in S/G2 arrested cells. P4D1 was validated through its recognition of recombinant di-ubiquitin. H4R3me2a was validated via in vitro methylation assay using recombinant PRMT1 and histone H4.

Confirmation of other antibodies are provided by the manufacture's website:
Phospho-Histone H3 Ser 10 (https://www.cellsignal.co.uk/products/primary-antibodies/phospho-histone-h3-ser10-antibody/9701)
Tubulin (https://www.sigmaaldrich.com/GB/en/product/sigma/t6199?context=product) actin https://www.sigmaaldrich.com/GB/en/product/sigma/a2228?context=product)
CENPF (https://www.cellsignal.co.uk/products/primary-antibodies/cenp-f-d6x4l-rabbit-mab/58982)
Mitosin https://www.bdbiosciences.com/en-gb/products/reagents/microscopy-imaging-reagents/immunofluorescence-reagents/purified-mouse-anti-human-mitosin.610768)
c-Myc (abcam) (https://www.abcam.com/myc-tag-antibody-9e10-ab32.html)
c-myc (Santa-Cruz) (https://www.scbt.com/p/c-myc-antibody-9e10?gclid=CjwKCAjw95yJBhAgEiwAmRrutI7F4T_fJtnMpOu2dzUawJ284cEm4G21ZXnATCod-vCajWfMLYkfDxoCg3QQAvD_BwE)
GFP(https://www.sigmaaldrich.com/GB/en/product/roche/11814460001?gclid=CjwKCAjw95yJBhAgEiwAmRrutDRX6YYlw1iqzlUkr5_8i7l8IneF0V5HxMZM--YHnqZpr7B2X7V6pBoC3dEQAvD_BwE)
HA (https://www.biolegend.com/en-us/products/anti-ha-11-epitope-tag-antibody-11071?GroupID=GROUP26)
GAPDH (https://www.abcam.com/gapdh-antibody-loading-control-ab9485.html).

Eukaryotic cell lines

Policy information about cell lines

Cell line source(s)	MCF7 (obtained from Gillian Farnies' laboratory but originally from ATCC), 293T and MDA-MB-231 (ATCC), HeLa (Grant Stewart's laboratory, University of Birmingham, but originally from ATCC). All derivatives of these cell lines were engineered in house.
Authentication	293T and MDA-MB-231 were authenticated by ATCC using STR profiling. HeLa and MCF7 cells were not authenticated.
Mycoplasma contamination	All cell lines were routinely tested for mycoplasma and were found to be negative (ELISA based assay).

Commonly misidentified lines (See ICLAC register)	No commonly misidentified cell lines were used in this study.

Flow Cytometry

Plots

Confirm that:

☒ The axis labels state the marker and fluorochrome used (e.g. CD4-FITC).

☒ The axis scales are clearly visible. Include numbers along axes only for bottom left plot of group (a 'group' is an analysis of identical markers).

☒ All plots are contour plots with outliers or pseudocolor plots.

☒ A numerical value for number of cells or percentage (with statistics) is provided.

Methodology

Sample preparation	We used flow cytometry to sort zsGreen positive cells to generate cell lines. Here, live cells were trypsinised and resuspended in FACS buffer and then send for sorting.<br><br>We also used FACS for G2/M checkpoint analysis. HeLa cells were treated with 5Gy IR and harvested at the time points indicated. After fixation in 70% ethanol, cells were washed twice in PBS (0.1% Tween-20) and then incubated on a roller at 4oC in ice-cold PBS (0.25% Triton-X 100) for 15 mins. After washing with PBS (1% BSA), cells were incubated in 100l PBS (1% BSA) containing 1:1000 dilution of phospho-histone H3S10 antibody (CST: 9701) for 1hr followed by a 1hr incubation with anti-rabbit DyLIGHT 594 secondary antibody (Fisher: 10108403). Cells were washed once with PBS (1% BSA), then PBS, and then resuspended in FACS buffer (PBS minus Ca2+; 2mM EDTA) supplemented with Vybrant DyeCycle Violet stain (Invitrogen: V35003). After 30 mins incubation at 37oC, cells were analysed by flow cytometry on a BD LSRFortessa X-20.<br><br>For cell cycle analysis (PI staining), cells were fixed in 4% PFA and incubated with prodidium iodide prior to analysis.
Instrument	BD LSR Fortessa X-20<br>BD FACS Aria
Software	BD FACS Diva software (v.8.0.1). Representative FACS profiles were generated using FlowJo (v10.6.1) software.
Cell population abundance	Percentage of positive cells depends on viral infection efficiency, routinely varying from 60-80%. Cell lines were routinely analysed by FACS to ensure maintenance of expression.
Gating strategy	Stable cell lines: parental cells were used as a negative control to set gates for zsGreen positive population. FCS/SSC gating was used to exclude cell debris. Gating strategies for G2/M checkpoint and cell cycle are presented in Supplementary Fig. 8.

☒ Tick this box to confirm that a figure exemplifying the gating strategy is provided in the Supplementary Information.
